# Targeting NFATc1-regulated MTHFD2 one-carbon metabolism to suppress sustained T-cell-mediated inflammation in rheumatoid arthritis

**DOI:** 10.1038/s41392-026-02752-y

**Published:** 2026-06-10

**Authors:** Theodora Manolakou, Jianyu Shen, Sanjaykumar Boddul, Martina Samiotaki, Michail Angelos Panagias, George Sentis, Tarcília Aparecida Silva, Alexandra Argyriou, Dionysis Nikolopoulos, Kumar Sanjiv, Karine Chemin, Fredrik Wermeling, Martin Henriksson, Ana Slipicevic, Per-Johan Jakobsson, Katerina Chatzidionysiou, Thomas Helleday

**Affiliations:** 1 Department of Oncology-Pathology, Science for Life Laboratory, Karolinska Institutet, Solna, Sweden; 2 Division of Rheumatology, Department of Medicine, Center for Molecular Medicine, Karolinska Institutet, Karolinska University Hospital, Stockholm, Sweden; 3 Institute for Bioinnovation, Biomedical Sciences Research Centre “Alexander Fleming”, Vari, Greece; 4 Clinical, Experimental Surgery & Translational Research, Biomedical Research Foundation of the Academy of Athens, Athens, Greece; 5 Department of Oral Surgery, Pathology and Clinical Dentistry, School of Dentistry, Federal University of Minas Gerais, Belo Horizonte, Brazil; 6 One-carbon Therapeutics AB, Stockholm, Sweden; 7 Department of Oncology and Metabolism, Medical School, Sheffield, UK

**Keywords:** Rheumatic diseases, Adaptive immunity

## Abstract

T cells are central drivers of inflammation across autoimmune and inflammatory diseases, yet current therapies inadequately target pathogenic T-cell pathways, limiting durable disease control. Here, we identified a novel, targetable transcriptional-metabolic axis that sustains inflammatory T-cell responses, characterized by NFATc1-regulated activation of MTHFD2-dependent one-carbon metabolism. We demonstrate that NFATc1 directly binds the *MTHFD2* promoter region, driving metabolic reprogramming in activated T cells from rheumatoid arthritis (RA) patients as well as in experimental arthritis models. Pharmacological inhibition of MTHFD1/2 using the novel small molecule TH9619 suppresses proinflammatory cytokine production, expands Foxp3⁺ regulatory T cells and protects against cartilage and bone damage in vivo. Proteomic profiling reveals that TH9619 elicits a distinct molecular response in patients’ T cells, divergent from the currently used anti-folate therapy, particularly in inadequate responders. These findings use RA as the proving ground to establish NFATc1-mediated MTHFD2 activation as a critical regulator of sustained T-cell-driven inflammation and support selective MTHFD1/2 inhibition as a novel, mechanism-based therapeutic strategy for RA.

## Introduction

T cells are essential orchestrators of immune responses, enabling host defense while maintaining tissue homeostasis. However, dysregulated T-cell activation and effector function can drive persistent inflammation, tissue destruction, and autoimmunity. Rheumatoid arthritis (RA), a chronic autoimmune disease affecting approximately 1% of the global population, exemplifies the consequences of aberrant T-cell activity, leading to persistent synovial inflammation, progressive joint destruction, and systemic complications if not treated early.^1^

Despite the availability of conventional synthetic, biological, and targeted synthetic disease-modifying antirheumatic drugs (DMARDs), including methotrexate (MTX) and tumor necrosis factor (TNF) inhibitors, a substantial fraction of patients exhibits inadequate or incomplete responses, and a significant minority develop treatment-refractory disease.^2,3^ These limitations stem from mechanisms of action of the drugs that do not exploit the disease characteristics and adverse effects, underscoring the urgent need for more precise therapeutic targets, personalized approaches and predictive biomarkers of treatment response.

In RA and other T-cell-driven autoimmune conditions, T cells are subjected to proliferative pressures and amplify their proinflammatory and tissue-invading behavior, aided by heightened folate and nucleotide production.^4^ Specifically, CD4^+^ T-cell subsets, including Th1, Th17, and peripheral helper T cells (Tph), infiltrate the synovium and produce proinflammatory cytokines such as IFNγ, IL-17, CXCL13 and TNFα.^5–9^ These cytokines amplify inflammation, promote cartilage degradation, and stimulate autoantibody production. Tph cells are proposed to support B-cell responses and autoantibody production, further exacerbating the disease.^8^ These T-cell subsets interact with other immune cells, including B cells and innate immune cells, forming a complex network that perpetuates the inflammatory response.

The imbalance between effector T cells and regulatory T cells (Tregs), whose suppressive functions can be undermined in inflammatory environments, further exacerbates immune dysregulation.^10^ However, the molecular mechanisms underlying sustained pathogenic T-cell activation and resistance to therapeutic suppression in chronic inflammation remain incompletely understood.

One-carbon (1C) metabolism is a key metabolic pathway supporting nucleotide synthesis, redox balance, and epigenetic regulation.^11–13^ Enzymes involved in one-carbon metabolism, such as SHMT1, SHMT2, MTHFD1, and MTHFD2, are reported to be induced upon T-cell activation.^14^ Among the enzymes involved in this pathway, methylenetetrahydrofolate dehydrogenase 2 (MTHFD2) has emerged as a potential regulator of T-cell function and inflammatory responses. MTHFD2 is upregulated in whole blood cells across various autoimmune diseases, including RA.^15^ This enzyme supports de novo nucleotide synthesis and regulates DNA methylation and histone modifications in effector T cells, particularly Th17 cells. Preclinical studies have demonstrated that targeting MTHFD2 reduces disease severity in multiple sclerosis disease models by promoting regulatory T-cell differentiation.^15^

MTX remains the cornerstone treatment for autoimmune arthritis, such as RA, and acts as a folate antagonist primarily through the inhibition of dihydrofolate reductase (DHFR) and other folate-dependent enzymes, ultimately interfering nonspecifically with one-carbon metabolism.^16–18^ Beyond DHFR, MTX also targets many more enzymes, such as ATIC and TYMS, contributing to the accumulation of adenosine and suppression of immune cell function. However, its broad disease unspecific mechanism of action affects healthy proliferating cells,^19,20^ contributing to systemic toxicity, and a considerable subset of patients develops inadequate responses or intolerance, necessitating treatment escalation.^21^ These challenges call for precise approaches to target disease-relevant T-cell metabolic pathways while sparing healthy tissues.

Growing evidence underscores the transcription factor NFATc1 (i.e. nuclear factor of activated T cells, cytoplasmic 1) as a central driver of T-cell activation and effector differentiation, yet its connection to metabolic reprogramming in chronic inflammation remains poorly defined.^22–24^ Here, we identified a transcriptional-metabolic axis wherein NFATc1 directly induces MTHFD2 expression, linking T-cell activation to sustained one-carbon metabolism during inflammation. By integrating transcriptomic and proteomic analyses of patient-derived T cells, mechanistic studies, and preclinical models of inflammatory arthritis, we demonstrate that selective pharmacological inhibition of MTHFD1/2 with the novel small molecule TH9619^25,26^ suppresses proinflammatory cytokine production and NF-κB-driven programs. This inhibition is associated with increased expression of regulatory T-cell-related markers and provides strong protection against cartilage and bone damage. Importantly, we show that MTHFD1/2 inhibition elicits distinct molecular effects compared to the standard anti-folate therapy, MTX, particularly in RA patients with inadequate MTX responses, and offers an innovative therapeutic alternative. Collectively, using RA as the proving ground, our findings establish NFATc1-regulated MTHFD2-dependent one-carbon metabolism as a critical regulator of pathogenic T-cell responses, providing a mechanistic foundation for targeted metabolic therapies to address persistent inflammation and structural joint damage in RA and potentially other T-cell-driven autoimmune diseases.

## Results

### Hyperactive MTHFD2-dependent one-carbon metabolism occurs in selective RA T-cell subsets

**Fig. 1:**
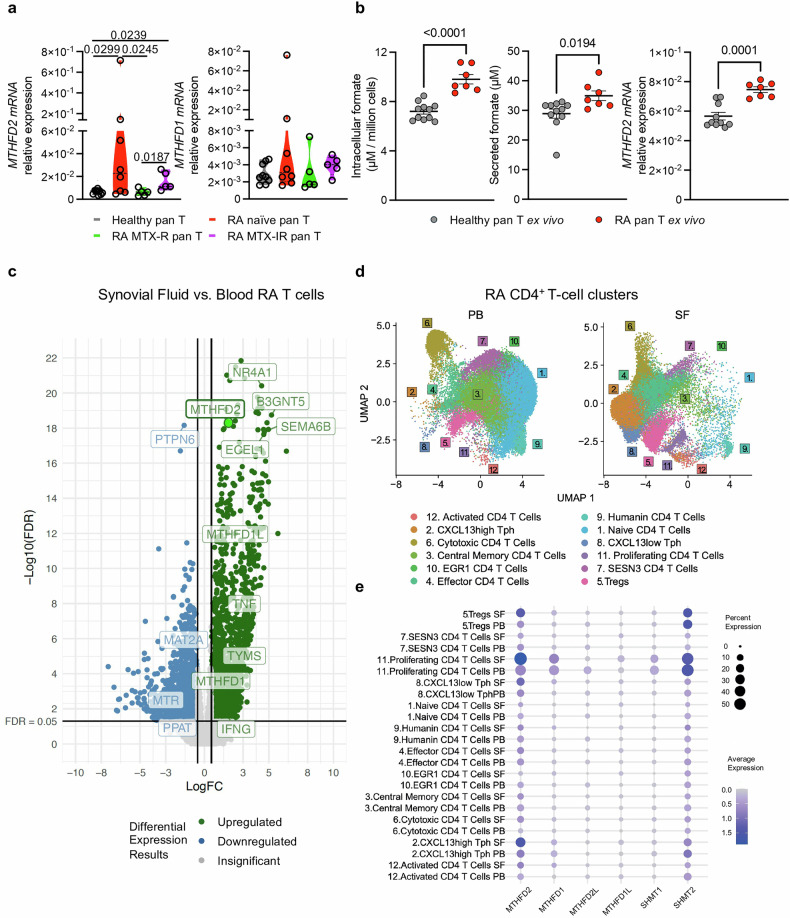
One-carbon metabolic flux and MTHFD2 are upregulated in RA T-cell subsets. **a** Relative expression of *MTHFD2* and *MTHFD1* mRNA in peripheral blood-derived pan T cells derived from healthy donors (*n* = 9), treatment-naïve (naïve, *n* = 8), MTX-responders (MTX-R, *n* = 5) and MTX-inadequate responders (MTX-IR, *n* = 5) RA patients, measured with real-time RT-qPCR and normalized to *ACTB*. T cells were purified using magnetic bead-based isolation. MTX-IR and MTX-R samples were collected post-MTX treatment. Data are shown as violin plots with individual data points. Statistical significance was obtained by the Kruskal-Wallis test followed by Dunn’s uncorrected test. *P* values < 0.05 are indicated in the respective graph. **b** Left and middle: Quantification of intracellular and secreted formate levels in pan T cells isolated from the peripheral blood of healthy donors (*n* = 11) and treatment-naïve RA patients (*n* = 7). T cells were purified using magnetic bead-based isolation and stimulated with CD3/CD28 beads for 24 h prior to colorimetric analysis. Right: Relative *MTHFD2* mRNA expression in the same samples, measured with real-time RT-qPCR and normalized to *ACTB*. For *MTHFD2* mRNA analysis, RNA was available for *n* = 10 healthy donors and *n* = 7 patients. Data are shown as scatter plots (mean ± SEM) with individual data points. Statistical significance was obtained by unpaired *t* test. *P* values < 0.05 are indicated in the respective graph. **c** Volcano plot of the differential expression analysis performed comparing synovial fluid (SF)-derived T cells of RA patients (*n* = 4) versus peripheral blood (PB)-derived T cells from treatment-naïve RA patients (*n* = 10) using a publicly available RNAseq dataset (GSE118829). Volcano plots focused on statistically significant genes (FDR < 0.05; |logFC| > 0.58). Selected one-carbon metabolism-related (e.g. *MAT2A, MTR, PPAT, MTHFD2, MTHFD1 MTHFD1L, TYMS*) and activation-associated (e.g. *NR4A1, TNF, IFNG*) genes are labeled. **d** UMAP displaying 12 CD4^+^ T-cell clusters in PB (left panel, *n* = 48,167 cells) and SF (right panel, *n* = 31,089 cells) from RA patients (*n* = 8). **e** Dot plot showing the expression of selected one-carbon metabolism-related genes (e.g. *MTHFD2*, *MTHFD1*, *MTHFD2L*, *MTHFD1L*, *SHMT1* and *SHMT2*) in the different CD4^+^ T-cell subsets in SF and PB. RA rheumatoid arthritis, MTX methotrexate, R responders, IR inadequate responders, PB peripheral blood, SF synovial fluid

We used RA as a disease model for T-cell-driven inflammation, given that T cells are central drivers of its pathogenesis and display heightened metabolic activity.^27–30^ To investigate whether MTHFD2 and other components of one-carbon metabolism are dysregulated in active RA, we first analyzed the transcript levels of *MTHFD2* and *MTHFD1* in pan T cells from RA patients who were treatment-naïve and from RA patients who had received methotrexate therapy (i.e. posttreatment samples), categorized as methotrexate-inadequate responders (MTX-IR) or methotrexate-adequate responders (MTX-R). Quantitative PCR (qPCR) analysis revealed that *MTHFD2* was significantly upregulated in peripheral blood-derived pan T cells from treatment-naïve and MTX-IR RA patients compared with healthy donors or MTX-R patients, whereas *MTHFD1* expression did not show any significant differences (Fig. 1a). This suggests that MTHFD2 may contribute to active immune flare-ups, during which T cells are highly immunogenic and cause disease. To validate MTHFD2 upregulation in T cells upon immunogenic activation, we stimulated ex vivo pan T cells isolated from healthy donors with CD3/CD28 beads. This resulted in increased levels of both mRNA and protein of MTHFD2, coinciding with markers of proliferation and activation (Supplementary Fig. 1a, b). Moreover, to confirm the link between one-carbon metabolism and RA, we assessed the protein levels of TYMS and ALDH1L1, which represent complementary branches of folate-dependent metabolism (Supplementary Fig. 2a, b). The upregulation of MTHFD2, MTHFD1, and TYMS, together with downregulated ALDH1L1 in treatment-naïve RA pan T cells compared with healthy controls, indicated a shift toward anabolic one-carbon flux that supports nucleotide synthesis and proliferation, suggesting enhanced engagement of folate-dependent one-carbon metabolism.^11^ We also observed significantly elevated intracellular and secreted formate levels, a direct product of mitochondrial one-carbon metabolism and a functional indicator of folate-mediated one-carbon flux^11^, together with increased *MTHFD2* mRNA levels in RA pan T cells compared with healthy T cells ex vivo (Fig. 1b), thereby validating our transcriptional and protein findings and confirming functional upregulation of one-carbon metabolism in RA T cells.

We next compared synovial fluid (SF)-derived T cells to peripheral blood (PB)-derived T cells from treatment-naïve RA patients using publicly available bulk RNA sequencing data (GSE118829). *MTHFD2* emerged as one of the most upregulated transcripts in SF-derived T cells (Fig. 1c), highlighting its potential role in T-cell responses within inflamed environments (i.e. arthritic joints). In addition to *MTHFD2*, other one-carbon metabolism-related genes (*MTHFD1*, *MTHFD1L*, *TYMS*) and activation-associated genes (*NR4A1, TNF, IFNG*) were significantly upregulated in SF-derived RA T cells, confirming heightened one-carbon metabolic activity and an activated T-cell state. Conversely, the downregulation of other one-carbon-linked metabolic and signaling genes, such as *MAT2A, MTR, PPAT* and *PTPN6*, in SF-derived T cells alongside high *MTHFD2* expression may suggest a selective rewiring of one-carbon metabolism, enabling functional adaptation in the inflamed joint microenvironment.

Therefore, to examine whether specific T-cell subsets validate this MTHFD2 signature in RA, we reanalyzed published single-cell RNA sequencing data of CD4^+^ T-cell clusters derived from SF and PB of RA patients (EGAS00001005241) for selected one-carbon metabolism gene expression (i.e. *MTHFD2*, *MTHFD1*, *MTHFD2L*, *MTHFD1L*, *SHMT1* and *SHMT2*) (Fig. 1d, e). Notably, proliferating, regulatory (Tregs) and CXCL13-high Tph cells, which were enriched in SF, exhibited elevated *MTHFD2* expression levels compared with other CD4^+^ T-cell clusters (Fig. 1d, e and Supplementary Fig. 3a, b). *MTHFD2* expression partially correlated with *SHMT2*, supporting enhanced one-carbon metabolism in these SF subsets (Fig. 1e).

To assess whether additional immune cell subsets exhibit dysregulated one-carbon metabolism in RA, we analyzed a publicly available microarray dataset (GSE93777) from various blood-derived immune cells of treatment-naïve RA patients and healthy donors focusing on the differential gene expression of key involved components, e.g. *MTHFD2*, *MTHFD1*, *MTHFD2L*, *AHCY*, *DHFR*, and *GART* (Supplementary Fig. 4a–f). The analysis showed that *MTHFD2* was profoundly upregulated in most T-cell subsets (i.e. CD4^+^ T effector memory cells, naïve CD4^+^ T cells and CD8^+^ T-cell subsets) from RA patients, consistent with our hypothesis that MTHFD2 may play a role in RA pathogenesis. While MTHFD2 expression was also detected in non-T-cell lineages such as monocytes and neutrophils, the differential expression between RA patients and healthy controls in these cell types was not significant compared with the robust upregulation observed in RA T cells (Supplementary Fig. 4a). This pattern highlights the T-cell-specific relevance of MTHFD2 in RA, consistent with the central role of T cells in driving autoimmune inflammation.

Overall, these data suggest a potential role for MTHFD2 in T-cell-mediated RA pathogenesis.

### Dysregulated one-carbon metabolism marks RA patients with poor treatment response

**Fig. 2:**
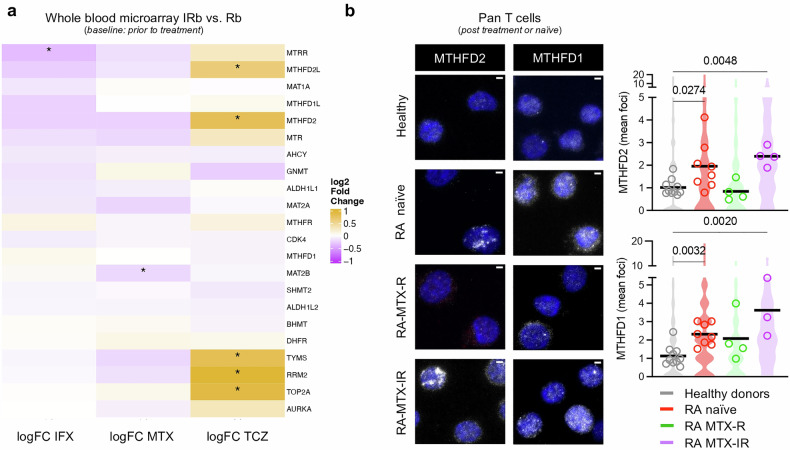
The MTHFD2-related molecular signature correlates to inadequate response to classical anti-folate therapy in RA patients. **a** Expression of one-carbon metabolism-related genes in the whole blood of RA patients using a publicly available microarray dataset (GSE93777). Expression values represent fRMA-normalized intensities. All samples (IRb and Rb) were collected prior to treatment initiation (b, baseline). The heatmap shows the log₂ fold change (IRb vs. Rb), with statistically significant differences annotated by asterisks. IFX infliximab (10Rb/9IRb); MTX methotrexate (15Rb/18IRb); TCZ tocilizumab (10Rb/3IRb). **b** Immunofluorescence microscopy of peripheral blood-derived pan T cells from RA patients (*n* = 8 treatment-naïve, *n* = 4 MTX-R, *n* = 3-4 MTX-IR, posttreatment) and healthy donors (*n* = 10) stained for MTHFD1 and MTHFD2 proteins. The results are presented as SuperPlots, where the circles represent the mean foci per individual, and the violin plots represent pooled foci across all individuals. Statistical significance was obtained by the Kruskal-Wallis test followed by Dunn’s uncorrected test. *P* values < 0.05 are indicated in the respective graph. Scale bar: 1 μm. RA rheumatoid arthritis, MTX methotrexate, R adequate responder, IR inadequate responder

We next wanted to test how MTHFD2-dependent one-carbon metabolism is differentially regulated in autoimmune patients with inadequate response to standard-of-care therapies, including MTX, infliximab (IFX, TNF inhibitor), and tocilizumab (TCZ, IL-6R blocker), prior to treatment initiation. To test this hypothesis, we analyzed the expression of *MTHFD2*, *MTHFD2L*, *MTHFD1*, and *MTHFD1L* in whole blood samples at baseline treatment (i.e. prior to treatment initiation) from RA patients who were later classified as responders or inadequate responders based on clinical outcomes^31^ using a publicly available microarray dataset (GSE93777). The results pinpointed a deregulation of one-carbon metabolism based on treatment response, with notable differences among treatments: TCZ inadequate responders showed upregulation of *MTHFD2* mRNA, while MTX and IFX inadequate responders exhibited downregulation of *MTHFD2* mRNA compared to their respective adequate responders **(**Fig. 2a**)**. Low baseline *MTHFD2* expression in patients likely indicates overall reduced one-carbon metabolic activity, which is further supported by the low expression of additional one-carbon metabolism genes such as *MTRR, MTHFD2L, MTR, MAT2A, MAT2B*, and *TYMS* (Fig. 2a).

Given MTX’s mechanism of action targeting components downstream of one-carbon metabolism and our previous mRNA findings on MTHFD2 in both treatment-naïve and posttreatment samples (Fig. 1a), we focused our subsequent functional investigation on posttreatment samples from patients who had received this common therapy. To this end, using immunofluorescence, we assessed MTHFD2 and MTHFD1 protein levels in pan T cells isolated from the PB of treatment-naïve, MTX-inadequate responders (MTX-IR), MTX-adequate responders (MTX-R), and healthy donors. Notably, we observed elevated protein levels of both MTHFD2 and MTHFD1 in treatment-naïve and MTX-IR RA patients compared to healthy donors, whereas MTX-R RA patients exhibited no significant differences from healthy donors (Fig. 2b). This may suggest that MTHFD2-dependent one-carbon metabolism is mitigated when inflammation is either absent (healthy donors) or effectively alleviated (MTX-R).

MTHFD2 protein expression correlated inversely with Disease Activity Score-28 (DAS28) in treatment-naïve samples (Supplementary Fig. 5), possibly because metabolically active MTHFD2- high T cells preferentially migrate to inflamed joints, reducing their representation in peripheral blood. This finding is consistent with the increased abundance of CXCL13-high Tph, Tregs, and proliferating CD4⁺ T cells in synovial fluid, subsets that express higher *MTHFD2* than their peripheral blood counterparts (Fig. 1e and Supplementary Fig. 3). MTX-IR patients displayed a similar but nonsignificant trend, while MTX-R samples did not show any evident trend.

Together, these findings indicate that dysregulation of MTHFD2-dependent one-carbon metabolism exists prior to treatment and may persist in patients with inadequate therapeutic response, whereas normalization of this pathway is associated with effective disease control.

### MTHFD1/2 inhibition suppresses pathogenic T-cell activity and overcomes anti-folate resistance ex vivo

**Fig. 3:**
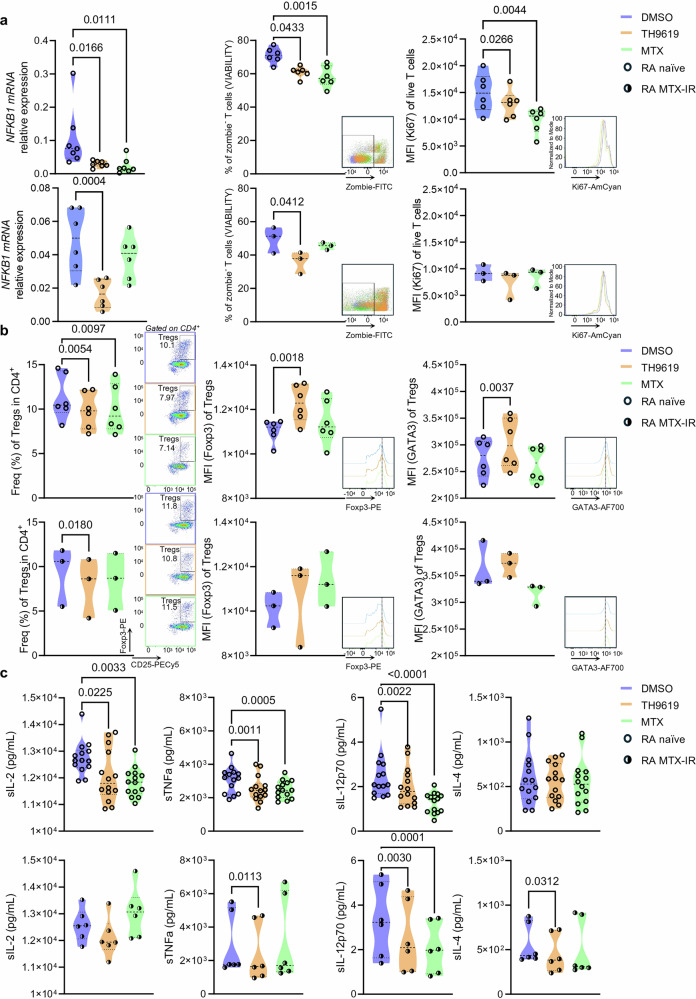
MTHFD1/2 inhibition suppresses pathogenic T-cell activity in RA ex vivo. Pan T cells were isolated from the peripheral blood of RA patients who were treatment-naïve (naïve, first row, *n* = 6-7) and MTX-inadequate responders (MTX-IR, second row, *n* = 3-6, posttreatment) using a magnetic bead-based approach and cultured with CD3/CD28 stimulatory beads in the presence or absence of MTHFD1/2i TH9619 (1 µM) or MTX (1 µM). DMSO was used as a vehicle. **a** Left: Relative expression levels of *NFKB1* mRNA measured with real-time RT-qPCR (normalized to*ACTB*) following culture of cells with the indicated compounds for 24 h. Middle: Viability rates of pan T cells as assessed by flow cytometry with Zombie staining following culture of cells with the indicated compounds for 24 h. Right: Assessment of proliferation as assessed by flow cytometry with Ki67 staining following culture of cells with the indicated compounds for 24 h. The cells analyzed for Ki67 were zombie-negative (viable cells). **b** Left: Flow cytometric analysis for the assessment of T regulatory cell frequency (Tregs: CD4^+^CD25^+^Foxp3^+^) following culture of cells with the indicated compounds for 24 h. Middle: Geometric mean fluorescence intensity (MFI) of the transcription factor Foxp3 within the Treg population. Right: Geometric MFI of the transcription factor GATA3 within the Treg population. All analyses were performed in zombie-negative cells (viable cells). **c** Detection of secreted cytokines (IL-2, TNFα, IL-12p70 and IL-4) using LEGENDplex technology following culture of cells with the indicated compounds for 65 h. For the Legendplex experiment, the results are depicted as *n* = 7 treatment-naïve biological replicates and *n* = 3 MTX-IR biological replicates in technical duplicates (7 × 2 and 3 × 2, respectively). For all cases, statistical significance was obtained by repeated measures one-way ANOVA followed by uncorrected Fisher’s least significance difference (LSD) test. Data are represented as violin plots showing all data points. *P* values < 0.05 are indicated in the respective graph. RA rheumatoid arthritis, MTX methotrexate, IR inadequate responder

We next examined whether targeting MTHFD2-dependent one-carbon metabolism can modulate autoimmune T-cell responses. The MTHFD1/2 inhibitor (MTHFD1/2i) TH9619, which selectively inhibits MTHFD1 and MTHFD2 (IC₅₀ 16 nM and 47 nM, respectively) over other folate-dependent enzymes (DHFR, TYMS, SHMT1 or SHMT2),^25,26^ was tested in CD3/CD28-stimulated T cells from treatment-naïve RA patients, MTX-IR RA patients and healthy donors. In treatment-naïve T cells, TH9619 markedly reduced *NFKB1* mRNA and multiple NF-κB-dependent mediators (*TNF, CXCL10, CCL20, and IL1B*) while leaving healthy donor T cells unaffected (Fig. 3a and Supplementary Fig. 6a). TH9619 also decreased viability (as determined by flow cytometric analysis of zombie-negative cells) and proliferation (Ki67) to an extent comparable to MTX (Fig. 3a). In MTX-IR T cells, DMSO controls exhibited intrinsically lower viability and proliferative capacity than treatment-naïve T cells, suggesting residual effects of prior MTX exposure and potentially increased susceptibility to TH9619. Under these conditions, TH9619 remained effective, suppressing *NFKB1, TNF*, and *CCL5* and reducing cell viability despite insignificant effects on *CXCL10, CCL20, IL1B* or proliferation (Fig. 3a and Supplementary Fig. 6a). Notably, TH9619 selectively reduced phosphorylated NF-κB p65 in MTX-IR, whereas MTX did not alter phosphorylated NF-κB p65 in either group (Supplementary Fig. 6b), indicating differential pathway susceptibility in MTX-IR cells.

TH9619 also affected regulatory and helper T-cell programs. While it reduced the overall Treg frequency in T-cell cultures, it increased Foxp3, GATA3, and CD86 expression within Tregs from treatment-naïve RA patients and upregulated GATA3 in CD4⁺ T cells, changes not observed with MTX, suggesting a shift toward a Th2-associated regulatory phenotype (Fig. 3b and Supplementary Fig. 7). These effects were not observed in MTX-IR T cells.

Furthermore, cytokine profiling revealed that both TH9619 and MTX reduced the proinflammatory cytokines IL-2, TNFα, and IL-12p70 in treatment-naïve cells, while IL-4 levels remained unaffected (Fig. 3c). Interestingly, in MTX-IR pan T cells, TH9619, but not MTX, decreased TNFα levels and affected IL-4 levels (Fig. 3c).

To confirm target specificity, we evaluated TH9619 responses in MTHFD2 knockout (KO) Jurkat T cells stimulated with CD3/CD28. In wild-type cells (WT), TH9619 suppressed proliferation (Ki67), activation (CD25), and phosphorylated NF-κB p65, whereas MTHFD2 KO cells showed no significant changes, demonstrating that TH9619’s effects on T-cell responses are specifically mediated through MTHFD2-dependent one-carbon metabolism targeting (Supplementary Fig. 8a–c).

Together, these findings show that MTHFD1/2 inhibition through TH9619 suppresses pathogenic T-cell activation, reprograms inflammatory signaling, and retains activity in MTX-IR T cells ex vivo, supporting TH9619 as a promising therapeutic strategy for RA.

### MTHFD1/2 inhibition rewires the RA T-cell proteome

**Fig. 4:**
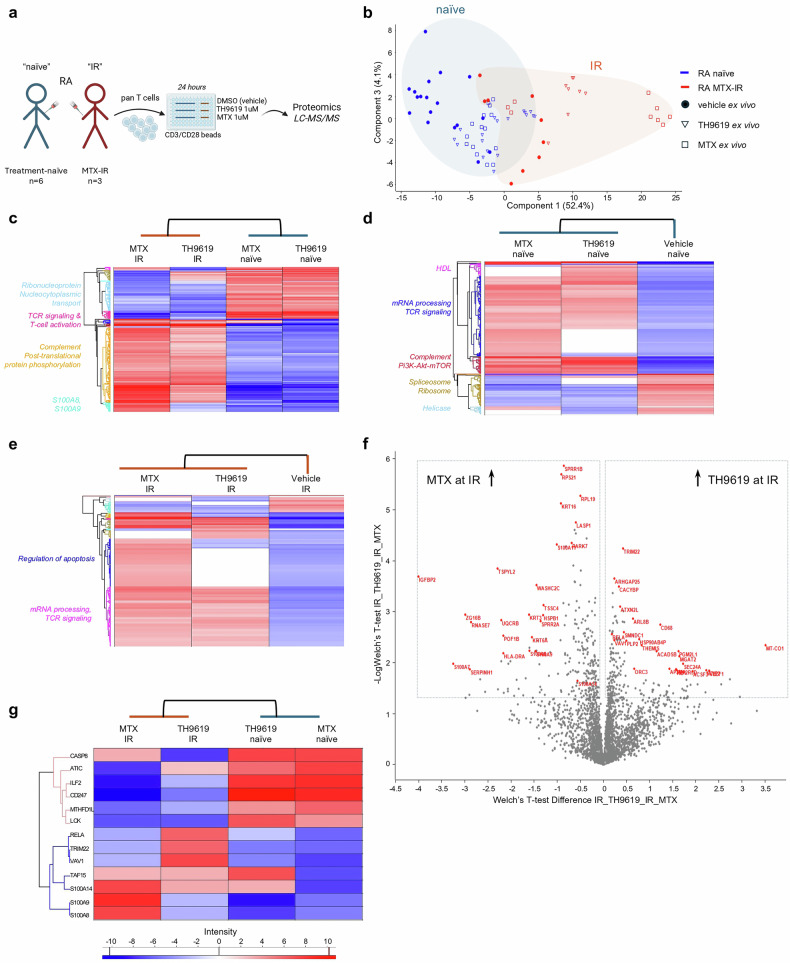
MTHFD1/2 inhibition rewires the T-cell proteome in RA distinctly from classical anti-folate therapy. **a** Pan T cells were isolated from the peripheral blood of treatment-naïve (naïve, *n* = 6) and MTX-IR (IR, *n* = 3) RA patients prior to incubation with CD3/CD28 stimulatory beads and treated for 24 h with TH9619 (1 µM), methotrexate (MTX, 1 µM), or DMSO (vehicle control). **b** Principal component analysis (PCA) based on proteins identified as significant by one-way ANOVA (*p* < 0.01), revealing distinct clustering of samples by treatment and responder status (*n* = 6 naïve and *n* = 3 IR in technical triplicates). **c**–**e** Heatmaps of ANOVA-significant proteins (using permutation-based FDR < 0.01), with *p* values derived from Tukey’s HSD post hoc test. The top enriched pathways for each cluster are indicated by the corresponding color on the left. **f** Volcano plot comparing TH9619 versus MTX treatments in MTX-IR T cells through a Welch’s *t* test (in red selected significantly altered proteins). **g** Heatmap of ANOVA-significant proteins (*p* < 0.01), with *p* values derived from Tukey’s HSD post hoc test. Selected proteins are indicated on the left. In all heatmaps, the first line of each column title indicates the ex vivo treatment (MTX, TH9619, or vehicle), while the second line denotes the prior clinical status: either inadequate response (IR) to the administered therapy or newly diagnosed (naïve) patients. RA rheumatoid arthritis, MTX methotrexate, IR inadequate responders

To characterize the molecular alterations induced by MTHFD1/2i TH9619 in RA T cells and to comprehensively compare them with those triggered by the classical anti-folate treatment, MTX, we conducted quantitative proteomics on ex vivo stimulated T cells from treatment-naïve and MTX-IR RA patients exposed to TH9619, MTX, or vehicle (Fig. 4a). Principal component analysis (PCA) of significant proteins exhibited a separation between treatment-naïve and MTX-IR samples, as well as distinct clustering of the three treatment conditions within each clinical group (Fig. 4b).

In treatment-naïve RA T cells, both TH9619 and MTX modulated ribonucleoprotein complexes, nucleocytoplasmic transport, TCR signaling, and T-cell activation pathways (Fig. 4c). Complement and posttranslational modification signatures were predominantly enriched in MTX-IR cells, indicating distinct stress-associated programs in the MTX-experienced compartment (Fig. 4c). Comparisons to untreated (vehicle) naïve RA T cells further showed that both treatments altered mRNA processing, TCR signaling, and PI3K-Akt-mTOR pathways (Fig. 4d) while exerting opposing effects on helicase-associated proteins and discoidal HDL-related clusters, highlighting mechanistic differences between TH9619 and MTX.

Proteomic signatures related to apoptosis differed markedly between the two treatments. In MTX-IR T cells, MTX increased the abundance of stress- and apoptosis-regulatory proteins, including COL2A1, P4HB, S100A9, VNN1, PSME3, PARK7, NOC2L, GSTK1, and RANGAP1 (Fig. 4e), consistent with apoptotic priming. In contrast, TH9619 markedly reduced cell viability (Fig. 3a), thereby potentially decreasing the representation of apoptosis-associated proteins in the proteomics dataset. These complementary readouts indicate that TH9619 may drive substantial cell loss, whereas MTX predominantly induces stress-responsive preapoptotic signaling. Interestingly, MTX uniquely increased S100A8/9 complexes in MTX-IR cells (Fig. 4f, g), a pattern consistent with inflammatory or cytotoxic responses associated with MTX exposure.^32^

We also pinpointed that while both TH9619 and MTX similarly modulated ATIC levels in treatment-naïve patients, only TH9619 affected ATIC expression in MTX-IR patients (Fig. 4g and Supplementary Fig. 9a). Moreover, MTHFD1L was selectively upregulated in treatment-naïve cells, irrespective of treatment conditions, consistent with metabolic distinctions between peripheral naïve and MTX-experienced T cells (Fig. 4g). Finally, in MTX-IR T cells, TH9619, but not MTX, upregulated key immune- and inflammation-associated proteins such as RELA (NF-κB p65), TRIM22 and VAV1 (Fig. 4f, g and Supplementary Fig. 9a, b), indicating differential modulation of these targets. The TH9619-associated increases in these proteins likely reflect compensatory or posttranslational regulation rather than enhanced inflammatory activity, consistent with the previous observation that TH9619 reduced *NFKB1* mRNA and multiple NF-κB target genes (Fig. 3a and Supplementary Fig. 6a), lowered phosphorylated NF-κB p65 at an early timepoint and did not alter it at a later timepoint despite an increase in total NF-κB p65 protein (Supplementary Figs. 6b and 9a).

These findings suggest that MTHFD1/2 inhibition through TH9619 may exert distinct immunomodulatory effects compared to MTX, particularly in MTX-IR RA patients, positioning it as a therapeutic candidate.

### MTHFD1/2i TH9619 ameliorates inflammation and protects against cartilage and bone damage in vivo

**Fig. 5:**
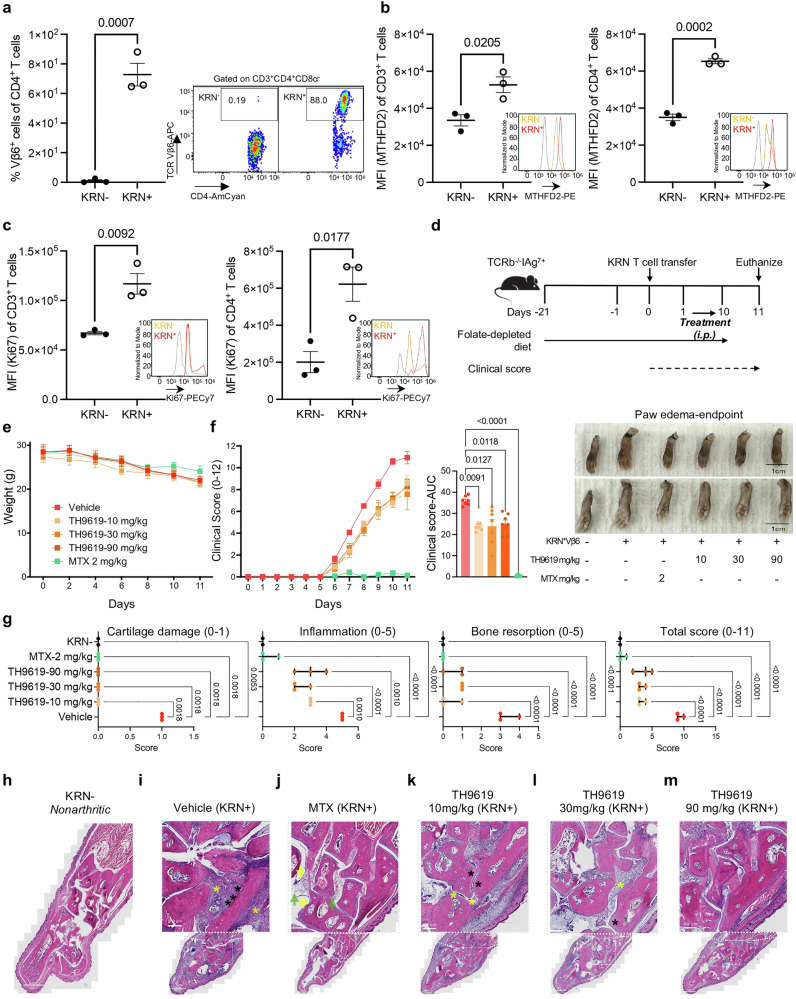
MTHFD1/2 inhibition reduces cartilage damage and bone erosion in a mouse model of inflammatory arthritis. The KRN T-cell transfer murine model was used to assess the effect of TH9619 MTHFD1/2i in inflammatory arthritis. MTX was used as a reference control for disease alleviation in this model. **a** Frequency of CD3^+^CD4^+^Vβ6^+^ T cells in the lymph nodes (LNs) of the mice. KRN^+^ indicates the mice that received CD3^+^CD4^+^Vβ6^+^ T cells and hence developed arthritis (*n* = 3 per group). Statistical significance was obtained by unpaired *t* test. **b** Protein expression levels of MTHFD2 depicted with geometric MFI in total T cells (CD3^+^) and T helper cells (CD4^+^) in the LNs derived from arthritic (KRN^+^) and nonarthritic (KRN^-^) mice as assessed via flow cytometry (*n* = 3 per group). Statistical significance was obtained by unpaired *t* test. In the representative histograms, orange represents MTHFD2 expression in KRN^-^ mice, red represents MTHFD2 expression in KRN^+^ mice, and gray indicates the background signal (negative control) generated by the anti-rabbit secondary antibody. **c** Assessment of proliferation as assessed by flow cytometry with Ki67 staining in total T cells (CD3^+^) and T helper cells (CD4^+^) in LNs derived from arthritic (KRN^+^) and nonarthritic (KRN^-^) mice (*n* = 3 per group). Ki67 intensity is depicted as geometric MFI. All flow cytometric analyses were performed in zombie-negative cells (viable cells). Statistical significance was obtained by unpaired *t* test. In the representative histograms, orange represents Ki67 expression in KRN^-^ mice, red represents Ki67 expression in KRN^+^ mice, and gray indicates the background signal (negative control) generated by an unstained sample. **d** Schematic representation of the in vivo studies. Briefly, mice were fed a folate-depleted diet for 21 days before KRN T-cell transfer and continued until the end of the experiment. Treatment with TH9619 (10, 30 or 90 mg/kg), MTX (2 mg/kg) or DPBS (vehicle) began one day after the T-cell transfer and was administered daily. **e** Daily body weight measurements of mice from the day of KRN T-cell transfer (day 0) to the study endpoint (day 11). **f** Left: Daily assessment of the joint score based on clinical criteria (*n* = 7 mice per group). Middle: Quantification of the total joint score based on the area under the curve of the graph on the left. Statistical significance was obtained by the Kruskal-Wallis test followed by Dunn’s uncorrected test. Right: Indicative photos of the murine paws upon the treatments at the endpoint. **g** Assessment of cartilage damage, inflammation, bone resorption and the total score based on H&E representative photos of the paws of the mice shown below (*n* = 2 KRN^-^/no treatment/healthy phenotype, *n* = 3 MTX 2 mg/kg (KRN^+^), *n* = 3 TH9619 10 mg/kg (KRN^+^), *n* = 3 TH9619 30 mg/kg (KRN^+^), *n* = 3 TH9619 90 mg/kg (KRN^+^) and *n* = 3 DPBS vehicle (KRN^+^). Data are represented as Min to Max plots showing all data points. Statistical significance was obtained by ordinary one-way ANOVA followed by uncorrected Fisher’s least significance difference (LSD) test except for the cartilage damage analysis, where zero standard error required use of the Kruskal-Wallis test. Histological H&E images of the nonarthritis group (**h**) and the arthritis groups (**i-m**) treated with the indicated compounds revealing the main histological structures of the paw joints. **i** Arthritic mice treated with vehicle (DPBS). Areas of bone resorption with osteoclasts are indicated with asterisks (*), and extensive inflammation in the articular tissues is indicated with yellow. **j** Arthritic mice treated with MTX (2 mg/kg). Yellow arrows show normal and continuous synovia. Green arrows show minimal inflammation in the subsynovial space. Inflammatory cells are not observed in the articular space. **k** Arthritic mice treated with TH9619 (10 mg/kg). Healthy cartilage and chondrocytes are indicated with black asterisks. The articular cavity is clean; however, there is evidence of some inflammatory cells (yellow asterisk). **l** Arthritic mice treated with TH9619 (30 mg/kg). Healthy appearance of cartilage and chondrocytes in the magnified image (*) and significant decrease in inflammation (in yellow). **m** Arthritic mice treated with TH9619 (90 mg/kg). There were no evident signs of inflammation in the joint cavity or cartilage damage. There is minimal inflammation in the periarticular tissues and no evidence of bone damage. Data are represented as the mean ± SEM. *P* values < 0.05 are indicated in the respective graph. Scale bars are shown on the images and correspond to 1 mm for the whole-joint views and 200 μm for the magnified regions. MFI geometric mean fluorescent intensity, LN lymph nodes, AUC clinical score area under the curve

To validate the therapeutic potential of MTHFD1/2 inhibition via TH9619 in T-cell-mediated RA, we employed the in vivo KRN T-cell transfer model (KRN-CTM) of T-cell-induced inflammatory arthritis. This model involves transferring KRN^+^ TCR transgenic T cells to genetically susceptible recipients that develop erosive arthritis within a week.^33^ The transferred KRN^+^ T cells were identified by their surface expression of Vβ6, confirming efficient engraftment (Fig. 5a). Consistent with our observations in human RA T cells, we found that MTHFD2 is overexpressed in T cells from arthritic mice (KRN^+^) (Fig. 5b), and these cells displayed high proliferative capacity^34^ (Fig. 5c). We then initiated TH9619 treatment according to the pipeline outlined in Fig. 5d. No significant changes in body weight were observed during the treatment period (Fig. 5e). A group of mice treated with MTX served as a reference control for disease alleviation in T-cell-dependent autoimmune arthritis in mice.^35^

**Fig. 6:**
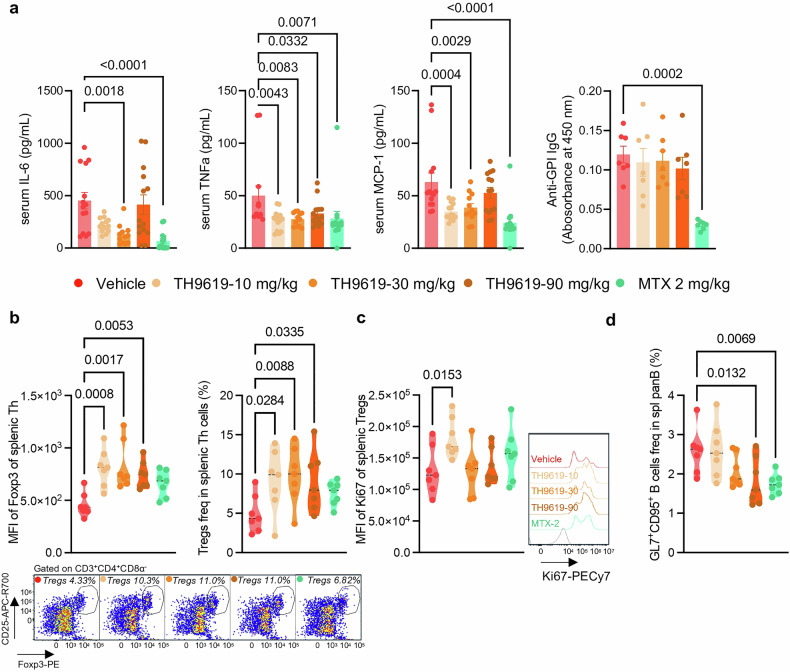
MTHFD1/2 inhibition reduces systemic inflammation and increases Tregs in a mouse model of inflammatory arthritis. The results shown here are based on the KRN T-cell transfer arthritis model and the experiment shown in Fig. 5. **a** Detection of cytokines (IL-6, TNFα and MCP-1) using LEGENDplex technology and anti-GPI IgG antibodies (ELISA) in the serum of treated arthritic mice (*n* = 7 per group). For the Legendplex experiment, the results are depicted as *n* = 7 biological replicates in technical duplicates (7 × 2). Data are represented as the mean ± SEM. **b** Flow cytometric analyses of murine splenic T cells upon the indicated treatments (*n* = 7 mice per group). The analyses show the geometric MFI of Foxp3 in T helper cells (CD4^+^) and the frequency of T regulatory cells (Tregs, CD4^+^CD25^+^Foxp3^+^) in the T helper cell population. The bottom panel below (**b**) shows the gated Tregs (CD4^+^CD25^+^Foxp3^+^) per group. **c** Assessment of proliferation as assessed by flow cytometry with Ki67 staining in splenic Tregs (*n* = 7 mice per group). Ki67 intensity is depicted as geometric MFI. In the representative histogram for Ki67 expression, gray indicates the background signal (negative control) generated by the FMO (fluorescence minus one, Ki67) sample. **d** The frequency of germinal center B cells (CD19^+^GL7^+^CD95^+^) in the murine spleen as assessed via flow cytometry (*n* = 7 mice per group). All flow cytometric analyses were performed in zombie-negative cells (viable cells). Data are represented as violin plots showing all data points. For (**b-d**), statistical significance was obtained by the Kruskal-Wallis test followed by Dunn’s uncorrected test. *P* values < 0.05 are indicated in the respective graph. MFI geometric mean fluorescent intensity, Th T helper cells (CD4^+^), spl spleen

Remarkably, mice treated with TH9619 across various dosages (10, 30, and 90 mg/kg) demonstrated significant joint score reduction, as evaluated by daily clinical criteria (Fig. 5f) and histopathological features (cartilage damage, inflammation, and bone resorption) (Fig. 5g–m). Therapeutic efficacy was comparable across all doses, indicating a plateau in efficacy that is consistent with saturation of the MTHFD2-dependent pathway at lower concentrations. Serum proinflammatory cytokines, including IL-6, TNFα, and MCP-1, were diminished upon TH9619 administration, particularly at lower dosages (10 or 30 mg/kg). However, glucose-6-phosphate isomerase (GPI)-specific autoantibodies were significantly decreased only in MTX-treated cells, consistent with the broader B-cell-modulatory effects of MTX (Fig. 6a). Notably, comprehensive immunophenotyping of splenic, lymphoid, and blood T and B-cell subsets (Fig. 6b, c, and Supplementary Figs. 10, 11) showed that TH9619 treatment increased both the frequency and Foxp3 expression levels of splenic Tregs. MTX produced a similar but nonsignificant trend (Fig. 6b). This increase correlated with enhanced Treg proliferative capacity at the lowest TH9619 dose (Fig. 6c), while overall CD4⁺ T-cell proliferation was also elevated at this dose (Supplementary Fig. 11). In parallel, germinal center (GC) B cells were partially reduced (Fig. 6d), suggesting that TH9619 may restrict B-cell pathogenicity through Treg-mediated regulation. In the lymph nodes or blood, MTX appeared more potent in reducing CD4^+^ T cells and GC B cells while also increasing Tregs (Supplementary Fig. 11).

To assess the potential off-target effects of TH9619, we profiled additional immune subsets in KRN-CTM mice, including monocytes, neutrophils, macrophages, dendritic cells, and NK1.1^+^ cells (Supplementary Fig. 12a–d). TH9619 induced only limited perturbations: an increase in circulating macrophages at the highest dose and a decrease in splenic NK1.1⁺ cells at 30 mg/kg, consistent with a treatment acting primarily on T cells. In contrast, MTX elicited widespread alterations across multiple lineages, including neutrophils, macrophages, dendritic cells, and NK1.1⁺ cells in the spleen as well as NK1.1⁺ cells in the blood.

Together, these data demonstrate that pharmacological MTHFD1/2 inhibition with TH9619 alleviates inflammation and provides robust protection against structural joint damage, with a narrower immune footprint than MTX.

### NFATc1 modulates MTHFD2 via promoter binding in RA T cells

To elucidate the molecular mechanisms driving MTHFD2 upregulation in autoimmune RA T cells, we first used the Eukaryotic Promoter Database (EPD), TFmotifView, and Gene Transcription Regulation Database to map candidate transcription factor-binding sites within the *MTHFD2* regulatory region. Among several predicted factors, NFATc1 has emerged as a strong candidate owing to its established roles in T-cell activation and RA-associated osteoclastogenesis.^36,37^

**Fig. 7:**
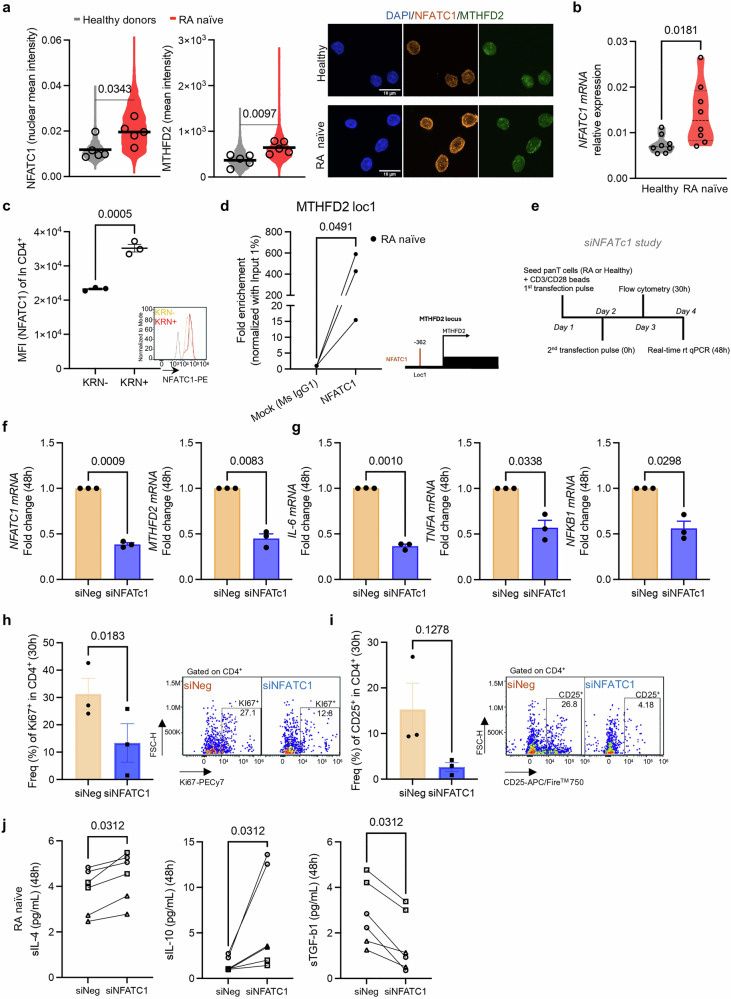
NFATc1 regulation of MTHFD2 determines pathogenic T-cell responses. **a** Immunofluorescence microscopy analysis of blood-derived pan T cells from healthy donors and treatment-naïve RA patients (*n* = 5 per group) for the fluorescence intensity of NFATc1 and MTHFD2 proteins. Measurements of NFATC1 represent its nuclear expression, as assessed by overlapping DAPI with the NFATC1 signal in CellProfiler. The results are presented as SuperPlots, where the circles represent the mean intensity per individual, and the violin plots represent the pooled MFI across all individuals. Statistical significance was obtained by unpaired *t* test. **b** Relative expression levels of *NFATc1* mRNA in pan T cells derived from the peripheral blood of healthy donors and treatment-naïve patients (*n* = 8 per group) measured with real-time RT-qPCR and normalized to *ACTB*. Data are represented as violin plots showing all data points. Statistical significance was obtained by unpaired *t* test. **c** Protein expression levels of NFATc1 depicted with geometric MFI in lymphoid T helper cells (CD4^+^) derived from arthritic (KRN^+^) and nonarthritic (KRN^-^) mice as assessed via flow cytometry (*n* = 3 per group). The gray curve in the histogram panel corresponds to an unstained sample. Data are represented as the mean ± SEM. Statistical significance was obtained by unpaired *t* test. **d** ChIP analysis of the binding site of NFATc1 to the *MTHFD2* gene locus (loc1) real-time quantitative PCR. ChIP experiments were performed using anti-NFATc1 antibody or a control antibody (IgG1) in chromatin isolated from CD3/CD28 ex vivo stimulated pan T cells derived from treatment-naïve RA patients (*n* = 3 RA patients; for each individual, NFATc1 expression was further normalized to the relevant mock control). Statistical significance was obtained by a ratio paired *t* test. **e** Schematic representation of silencing NFATc1 expression in CD3/CD28-stimulated pan T cells derived from treatment-naïve RA patients using siNFATc1 or siNeg (control). **f**, **g** Relative expression levels of *NFATc1, MTHFD2, IL6, TNF* and *NFKB1* mRNA in siNFATc1 and siNeg conditions measured with quantitative real-time RT-qPCR (*n* = 3 treatment-naïve RA patients). Data are represented as the mean ± SEM. Statistical significance was obtained by paired *t* test. **h**, **i** siNFATc1 and siNeg RA T cells were stained and analyzed for proliferation (Ki67) and activation (CD25) of the T helper (CD4^+^) population via flow cytometry (*n* = 3 treatment-naïve RA patients). Data are represented as the mean ± SEM. Statistical significance was obtained by paired *t* test. **j** Detection of cytokines (IL-4, IL-10 and TGF-β1) using LEGENDplex technology in the cell culture supernatants of the siNFATc1 and siNeg RA T cells at the endpoint (*n* = 3 treatment-naïve RA patients). For the Legendplex experiment, the results are depicted as *n* = 3 biological replicates in technical duplicates (3 × 2). Statistical significance was obtained by the paired Wilcoxon test. *P* values are indicated in the respective graph. RA rheumatoid arthritis, MFI geometric mean fluorescent intensity, ChIP chromatin immunoprecipitation, loc locus

NFATc1 protein expression in the nucleus was significantly elevated in T cells from RA patients compared to healthy T cells, consistent with its transcriptional activation in fresh RA T cells, concurrent with upregulation of MTHFD2 mRNA and protein (Figs. 1a and 7a, b). Similarly, NFATc1 and MTHFD2 protein levels were increased in T cells from arthritic mice relative to nonarthritic controls (Figs. 5b and 7c).

In SF RA T cells, NFATC1-associated genes (*NR4A1, TNF, IFNG*) were also increased compared to blood T cells (Fig. 1c). The scRNA-seq data further revealed that *NFATC1* and its target genes (*CDK4, CD40LG, TNF, IFNg* and *NR4A1*)^38^ are differentially expressed across T-cell subsets. SF-derived T cells showed higher expression of these genes predominantly in CXCL13-high Tph cells, effector CD4⁺ T cells, proliferating CD4⁺ T cells, and Tregs (Supplementary Fig. 13), with partial overlap with *MTHFD2* (Fig. 1e, and Supplementary Fig. 3b). In contrast, PB-derived T cells exhibited increased *NFATC1* and target gene expression mainly in central memory and naïve CD4⁺ T-cell subsets (Supplementary Fig. 13).

To test whether NFATc1 directly binds the MTHFD2 locus, we performed ChIP assays with anti-NFATc1 antibody or control IgG1 in RA T cells activated ex vivo (Supplementary Fig. 14a, b). NFATc1 showed specific enrichment at a promoter-proximal region (loc1) adjacent to the transcription start site (Fig. 7d). Additional predicted sites were evaluated (Supplementary Fig. 14c), and binding appeared more pronounced in older donors in a limited dataset (*n* = 4), suggesting a potential age-related trend (Supplementary Fig. 14d**)**.

We next examined whether NFATc1-MTHFD2 interactions are functionally regulated. TH9619 reduced *NFATc1* and *MTHFD2* mRNA expression in RA T cells while sparing healthy donor T cells (Supplementary Fig. 15a) and diminished NFATc1 occupancy at the *MTHFD2* promoter-proximal region in stimulated Jurkat T cells (Supplementary Fig. 15b, c). Pharmacologic blockade of NFATc1 nuclear translocation with FK506 or cyclosporin A similarly reduced *NFKB1* expression and its target genes, mirroring TH9619’s downstream effects (Supplementary Fig. 16a, b).

To directly assess the regulatory role of NFATc1 binding on the *MTHFD2* gene locus, we performed knockdown of NFATc1 using small interfering RNA (siNFATc1) in RA T cells (Fig. 7e). NFATc1 silencing reduced *MTHFD2* expression (Fig. 7f), decreased *NFKB1* and associated proinflammatory genes (*IL6, TNF, IFNG*), reduced the proliferation capacity of the cells, and partially attenuated their activation status (Ki67, CD25, *IL2RA*, *IL2*) (Fig. 7h, i and Supplementary Fig. 17). In contrast, in healthy activated T cells, NFATc1 knockdown reduced *MTHFD2* mRNA but did not affect *NFKB1* and its target genes, suggesting that NFATc1 may regulate MTHFD2 in activated healthy T cells without influencing NF-κB signaling (Supplementary Fig. 17). Furthermore, NFATc1 knockdown in RA T cells increased the anti-inflammatory cytokines IL-4 and IL-10 while decreasing pathogenic TGF-β1 secretion (Fig. 7j).

Overall, these results support the notion that NFATc1 directly regulates MTHFD2 activity in RA T cells, promoting their inflammatory and proliferative responses while sparing activated healthy T cells, highlighting a potential pathway for targeted therapy with minimal impact on physiologic immune function.

## Discussion

Our data define a disease-specific transcriptional-metabolic circuit in RA in which NFATc1 directly binds and activates MTHFD2, sustaining one-carbon metabolism in pathogenic T cells. Here, we describe a previously uncharacterized NFATc1→MTHFD2 axis in patient-derived T cells, demonstrate selective suppression of NF-κB-driven programs by the MTHFD1/2 inhibitor TH9619 in RA T cells sparing healthy T cells, and show robust in vivo protection against cartilage and bone damage with a narrower immune footprint than MTX.

The upregulation of MTHFD2 likely reflects the increased demand for nucleotide synthesis during T-cell activation, especially in CD4⁺ subsets, highlighting how autoimmunity relies not only on immune hyperactivity but also on metabolic reprogramming that sustains chronic inflammation. Although macrophage-driven inflammation within inflamed tissues, such as the synovium, has dominated RA paradigms, the efficacy of abatacept in delaying synovitis (ARIAA, APIPPRA trials) underscores the therapeutic potential of selective T-cell modulation.^27,28^

TH9619 selectively targets MTHFD2-dependent one-carbon flux through a unique mechanism of action that is well described.^26^ Briefly, it targets the MTHFD2 pathway by forming a disease-specific folate trap dependent on MTHFD2 activity and MTHFD1 inhibition.^26^ The effects of TH9619 are on-target, as MTHFD2^-/-^ cells show no response compared with wild-type cells (Supplementary Fig. 8). Furthermore, extensive profiling has identified no off-target binding beyond MTHFD1/2/2L,^25^ likely due to the compound’s high water solubility and limited potential for nonspecific hydrophobic interactions. In RA T cells (treatment-naïve or MTX-IR), TH9619 reduced *NFKB1* mRNA and several NF-κB target genes and lowered phosphorylated NF-κB p65 after short-term exposure. Although total NF-κB p65 protein increased later, its phosphorylation did not, suggesting compensatory accumulation rather than activation, a pattern consistent with transient NF-κB inhibition rather than sustained proinflammatory signaling.

Proteomic profiling revealed that TH9619 and MTX both modulate T-cell pathways involved in mRNA processing, ribonucleoprotein metabolism, and TCR signaling but diverge in apoptotic and stress responses. MTX-treated T cells likely remained viable and displayed increased abundance of stress- and apoptosis-regulatory proteins, whereas TH9619 likely reduced the viable population, lowering the detectable representation of apoptosis-associated proteins in the proteomic dataset.

In vivo, TH9619 treatment ameliorated arthritis in the KRN T-cell transfer model, reducing inflammation, cartilage damage, and bone resorption, consistent with prior reports implicating MTHFD2 in arthritic joint pathology.^39^ Efficacy was comparable across doses, indicating a plateau in pharmacodynamic response once the biological effective dose (BED) had been reached. This is consistent with our pharmacokinetic data^25^ showing that even the lowest tested dose (10 mg/kg) achieves plasma concentrations above the effective range. TH9619 lowered serum IL-6, TNFα, and MCP-1 levels, while MTX uniquely reduced pathogenic GPI autoantibodies, reflecting broader B-cell modulatory effects. TH9619 increased splenic Treg frequency and Foxp3 expression without major myeloid perturbation, whereas MTX affected multiple lineages, consistent with multitarget antifolate activity, a pattern that aligns with the wider clinical toxicity profile and dose-limiting side effects associated with MTX therapy. While TH9619 matched or outperformed MTX in some readouts, it did not fully replicate MTX’s broad anti-inflammatory efficacy in vivo. This apparent difference in efficacy must be interpreted with caution; as we have previously shown,^26^ the activity of the MTHFD1/2 inhibitor TH9619 is markedly reduced in mice due to high thymidine and low hypoxanthine levels compared with humans. This is a metabolic environment that suppresses TH9619 activity but does not affect MTX. Consequently, the murine model is intrinsically favorable for MTX and challenging for TH9619, and the observed activity of TH9619 likely underestimates its human potential. Our in vivo data should therefore be viewed as proof-of-concept for metabolic targeting rather than a direct potency comparison with MTX.

At the molecular level, NFATc1 directly binds the *MTHFD2* promoter-proximal region, and its knockdown in RA T cells reduces MTHFD2 expression, proliferation, and NF-κB target genes expression while enhancing IL-4 and IL-10 secretion. These findings identify NFATc1 as an upstream regulator linking T-cell activation to metabolic reprogramming and establish MTHFD2 as a pivotal node for inflammatory signals.

Low baseline (i.e. prior to treatment) MTHFD2 in MTX- or IFX-inadequate responders may reflect a less metabolically active T-cell compartment with limited engagement of one-carbon metabolism, potentially explaining their poor response to therapies that rely on dampening proliferative or metabolically primed immune cells. In contrast, high baseline MTHFD2 levels in TCZ-inadequate responders may suggest a metabolic phenotype in their T cells that supports persistent one-carbon flux and redox capacity, potentially maintained by IL-6-STAT3 signaling.^40^ Because these samples were examined before therapy, the elevated MTHFD2 likely predicts a subgroup with ongoing metabolic activity and disease biology less likely to be altered by subsequent IL-6R blockade. Clinically, such persistent metabolic activity highlights a subgroup whose disease biology may remain dependent on MTHFD2, raising the possibility that these patients could benefit from targeted inhibition of one-carbon metabolism. The persistent elevation of MTHFD2 after MTX treatment further underscores its involvement in sustaining chronic inflammatory signaling, supporting baseline MTHFD2 as a candidate biomarker for decision-making toward precision medicine.

Pharmacological MTHFD1/2 inhibition with TH9619 reduced T-cell activity and proinflammatory cytokine production in both treatment-naïve and MTX-resistant RA T cells while enhancing Treg-associated markers in treatment-naïve RA T cells. The observed differences between RA treatment-naïve and MTX-IR T cells upon TH9619 treatment may reflect residual effects of prior MTX exposure, which may influence their responsiveness to MTHFD1/2 inhibition. Proteomic analysis confirmed anti-inflammatory signatures with context-specific adaptations: immunomodulatory suppression in naïve cells and metabolic feedback regulation in MTX-IR cells. Overall, MTHFD1/2 inhibition modulated effector and regulatory T-cell-related programs in both settings, suggesting potential applicability in treatment-naïve and MTX-refractory RA, particularly during metabolically active inflammatory states.

To place TH9619 in the context of other MTHFD2-targeted approaches, we have previously compared it with another inhibitor tested in vivo. The DS18561882 compound was reported as a selective low-nM MTHFD2 inhibitor,^41^ yet in our assays, it inhibited both MTHFD1 and MTHFD2 at micromolar potency (3.0 µM and 5.6 µM, respectively).^25^ Nonetheless, it reduced inflammation in a T-cell-dependent delayed type hypersensitivity (DTH) model at 100 mg/kg,^15^ supporting the feasibility of pharmacologically disrupting MTHFD2 activity in vivo. TH9619 works through a distinct mechanism, as it does not enter mitochondria and only indirectly targets MTHFD2 activity via a folate trap through MTHFD1 inhibition.^26^ Together, we can conclude that both direct and indirect targeting of MTHFD2 can modulate pathogenic T-cell responses. The jury is out with respect to which strategy is the most efficient and will likely be determined in clinical trials, which have already started with TH9619, initially in cancer indications (NCT07151040).

Mechanistically, we established MTHFD2 as a transcriptional target of NFATc1, a key regulator of T-cell function.^22,42,43^ As NFATc1 is activated downstream of TCR signaling via calcium-dependent dephosphorylation, TH9619 may indirectly modulate NFATc1 activity, potentially by altering calcium flux^42,44,45^ or influencing chromatin accessibility at the MTHFD2 locus, limiting NFATc1 recruitment.^46^ This highlights the potential for a dual strategy to modulate both T-cell activation pathways and metabolic reprogramming in autoimmunity. Future studies employing calcium imaging and ATAC-seq will clarify these mechanisms. Notably, modulating NFATc1 activity, for example, by preventing posttranslational modifications such as SUMOylation, can promote Treg expansion and ameliorate experimental autoimmune disease models.^37^

While selective inhibition of MTHFD2-dependent one-carbon metabolism by TH9619 appears to promote regulatory phenotypes, the long-term impact on immune homeostasis and tissue-resident T cells remains to be defined. Integrating metabolomic analyses of TH9619-treated T cells will be critical to connect transcriptional and protein changes with functional and phenotypic outcomes. Future work will validate these findings in larger patient cohorts and delineate the molecular pathways connecting MTHFD2 activity to proinflammatory cytokine regulation. The differential metabolic environment in mice compared to humans also represents a major barrier for the interpretation of the human efficacy of this treatment.

In conclusion, these findings establish NFATc1-regulated MTHFD2 activation as a key driver of pathogenic T-cell metabolism in RA and demonstrate that pharmacological MTHFD1/2 inhibition can attenuate autoimmune inflammation while preserving immune balance (Supplementary Fig. 18-working model). By demonstrating the feasibility of pharmacologically targeting MTHFD2-dependent one-carbon metabolism to recalibrate immune responses, we propose a novel therapeutic strategy for RA that may extend to other T-cell-driven autoimmune diseases.

## Materials and methods

### Human subjects

This study was approved by the research ethics committee of Stockholm (ethical permit no. 2009/805-31/4), and all patients signed informed consent according to the Declaration of Helsinki. All patients had a clinical diagnosis of RA, and all of them fulfilled even the ACR/EULAR 2010 classification criteria. Peripheral blood was collected from 33 patients with RA, including 20 treatment-naïve individuals at the time of diagnosis, 5 MTX responders defined as achieving remission at 3, 6 or 12 months of follow-up as assessed by the treating rheumatologists and 8 MTX-inadequate responders defined as moderate or high inflammatory activity up by the treating rheumatologists despite at least 3 months of MTX treatment. Samples were collected at several visits at the Rheumatology Clinic of Karolinska University Hospital, Stockholm (Supplementary Table S1). Healthy donor blood samples were collected from Karolinska University Hospital blood centers (*n* = 27). PBMCs were isolated by Ficoll separation (GE Healthcare) and were used either fresh for pan T-cell isolation with EasySep (EasySep™ Human T-Cell Isolation Kit, Cat. 17951, STEMCELL) or cryopreserved in CryoStor™ CS10 (STEMCELL) for later pan T-cell isolation.

### KRN T-cell transfer model (KRN-CTM)

Twelve- to sixteen-week-old male and female KRN TCR transgenic B6. KRN (I-Ab/I-Ab) and B6xNOD TCRb^−/−^ (I-Ab/I-Ag7) mice were used in all experiments. The B6. The KRN strain was kindly provided by Diane Mathis and Christophe Benoist (Harvard Medical School).^47^ The B6 TCRb^−/−^ (stock no. 002118) and NOD (stock no. 001976) mouse strains were obtained from Jackson Laboratory, while the B6xNOD TCRb^−/−^ (I-Ab/-Ag7) strain was generated in-house by crossing B6 TCRb^−/−^ and NOD TCRb^−/−^ mice. All experiments were performed in accordance with ethical guidelines for humane animal use and were approved by the Regional Ethical Committee of Stockholm, Sweden.

The KRN T-cell transfer model of arthritis was employed as previously described.^33^ Briefly, 200 µL of splenocytes from B6. KRN mice were suspended in PBS and intravenously injected into the tail vein of B6 x NOD TCRb^−^^/^^−^ recipient mice. Disease progression was monitored by assessing body weight and joint swelling. Starting on the day after T-cell transfer (Day 1), mice received daily intraperitoneal (IP) injections of TH9619 (10, 30, or 90 mg/kg), MTX (2 mg/kg), or vehicle (DPBS) for 10 consecutive days (Days 1–10), as described in the scheme. Mice were euthanized on day 11 for organ collection. Mice were fed a folate-depleted diet (formula TD.01013, Inotiv-Teklad) to approximate human physiological folate levels. Murine plasma concentrations of 5-methyltetrahydrofolate (5-MTHF) are naturally much higher than those in humans and would otherwise mitigate the folate-trap mechanism through which TH9619 acts.^26^ No significant changes in body weight were observed throughout the study, indicating the absence of overt toxicity associated with the treatments (Fig. 5e) or the continuous folate-depleted diet (Supplementary Fig. 12a). Scoring of the arthritis model was performed in a blinded manner. Each limb was assessed on a 0–3 scale: 0 = no observable swelling; 1 = one affected digit or mild swelling of the foot/ankle with preservation of the original V shape; 2 = swelling resulting in parallel long edges of the foot with loss of the V shape; 3 = inversion of the V shape due to expansion of the ankle and hindfoot beyond the width of the forefoot. The scores from all four limbs were then summed to generate the clinical index, with a maximum score of 12 points.^48^

#### Preparation of tissues (paws/joints) for histology

Prior to hematoxylin and eosin (H&E) staining, the paws were fixed in 4% PFA for 24 h and then decalcified in a 15% EDTA solution for three weeks at 4 °C, with the solution being changed every other day. The samples were then washed in tap water, stored in 70% ethanol, and subsequently processed for standard H&E staining and scanning at the Morphological Phenotype Analysis Core Facility (FENO) of Karolinska Institutet. Joints were scored for inflammation, cartilage damage and bone resorption, as previously described,^33^ by a pathologist blinded to group status (T.A.S.).

### Cell culture

Pan T cells were isolated from frozen PBMCs using the EasySep Human T-cell isolation kit and cultured at 37 °C in a humidified incubator with 5% CO_2_ while plated in a 96-well round-bottom plate at a concentration of 1–2 × 10^5^ cells per well. Cells were cultured in Human Plasma-Like Medium (HPLM, Gibco, Cat. A4899101) supplemented with 10% (v/v) dialyzed FBS (Gibco, Cat. A3382001), penicillin-streptomycin (100 U/ml and 100 μg/ml, respectively; Gibco, Cat. 15070063), sodium pyruvate (1 mM; Gibco, Cat. 11360070), HEPES (10 mM; catalog no. 15630106, Gibco), and 2-mercaptoethanol (0.05 mM; Gibco, Cat. 31350010). In addition, for T-cell stimulation, cells were administered Dynabeads Human T-Activator CD3/CD28 (Gibco, Cat. 11131D) according to the manufacturer’s instructions.

Jurkat cells were purchased from ATCC. They were maintained in RPMI1640 medium with GlutaMax supplemented with 10% (v/v) dialyzed FBS and penicillin-streptomycin (100 U/ml and 100 μg/ml, respectively). Cells were maintained at 37 °C and 5% CO_2_ in a humid incubator.

### RNA-seq and microarray data

#### Bulk RNA sequencing data analysis

RNA sequencing data of RA patients and healthy controls were downloaded from the GEO database (GEO accession: GSE118829). Counts were TMM-normalized, and normalized expression of isolated T cells from RA patient (*n* = 4) synovial fluid samples and treatment-naïve RA patient (*n* = 10) blood samples were selected for subsequent differential expression analysis using the edgeR R package (v3.38.1).^49^ Differential expression results were considered statistically significant when FDR < 0.05 and |logFC| > 0.58. Volcano plots were created using the ggplot2 R package (v3.4.1).^50^

#### Microarray data analysis

Transcriptomic microarray data of RA patients were downloaded from the GEO database (GEO accession: GSE93777). Data of sorted immune cell types were visualized using ggplot2 to explore the expression of genes of interest. Whole blood data were used to perform differential expression analysis between treatment-baseline samples of RA patients defined as inadequate responders (IRb) vs. responders (Rb) to each treatment, namely, methotrexate (MTX), tocilizumab (TCZ) or infliximab (IFX), as defined in the original publication.^31^ The expression data were processed using fRMA normalization (Bioconductor, default settings) against internal reference arrays, rather than healthy controls. Downstream of normalization, the fRMA normalized matrix was used as input to the MEAL package runPipeline function for differential expression analysis. Patients were characterized as responders if they showed an adequate clinical response between weeks 2 and 36 to IFX, weeks 3 and 27 to TCZ, and weeks 4 and 28 to MTX. Heatmaps visualizing the results were created using the ComplexHeatmap R package (v2.18.0).^51^

#### Single-cell RNA-seq (scRNA-seq)

*MTHFD2* expression was analyzed in CD4^+^ T cells from paired peripheral blood and synovial fluid samples of eight RA patients (4 ACPA^−^, 4 ACPA^+^) as part of a broader single-cell RNA sequencing dataset.^7^ The dataset was analyzed in R (version 4.3.1), and the published clustering was reproduced using Seurat (version 5.0.1).^52^

### Proteomics

#### Sample preparation

Immunomagnetic negative isolation was used to obtain untouched human T cells from previously frozen PBMC samples (stored in CryoStor CS10 at -150 °C) of treatment-naïve (*n* = 6) and MTX-IR (*n* = 3) RA patients (Supplementary Table S1) following deoxyribonuclease treatment of the PBMCs. The isolated T cells were then cultured and stimulated in 96-well round-bottom plates for suspension cells at a concentration of 1.5 × 10^5^ cells per well in full HPLM and CD3/CD28 Dynabeads as described above in the presence of TH9619 (1 µM), MTX (1 µM) or DMSO (vehicle control) for 24 h. After incubation, the cells were washed twice with DPBS, pelleted, snap-frozen, and stored for subsequent lysis and processing.

#### Sp3-mediated protein digestion

The isolated T cells were lysed in 30 µl of lysis buffer consisting of 4% SDS, 100 mM DTT and 100 mM triethylammonium bicarbonate (TEAB). The mixtures were subjected to heating at 99 °C for 5 min and water-bath mediated sonication. Finally, the samples were centrifuged at 17,000 × *g* for 15 min, after which the supernatant was processed according to the Single-Pot Solid-Phase-enhanced Sample Preparation (Sp3) method of Hughes,^53^ omitting the acidification step and incorporating cysteine alkylation with 100 mM iodoacetamide. Digestion was performed at 37 °C with continuous agitation (1200 rpm) using 0.5 μg Trypsin Platinum (Promega) in 100 mM TEAB buffer. The next day, the magnetic beads were removed, and the peptidic samples were further purified by Sp3 peptide cleanup and evaporated to dryness in a vacuum centrifuge. The dried samples were solubilized in Buffer A and sonicated for 5 min, and the peptide concentration was determined by measuring the absorbance at 280 nm using a NanoDrop spectrophotometer.

#### LC-MS/MS analysis

Samples were analyzed by liquid chromatography-tandem mass spectrometry (LC-MS/MS) using a Dionex Ultimate 3000 RSLC system with a Thermo Q Exactive HF-X Orbitrap mass spectrometer. Peptidic samples were directly loaded onto a 25 cm-long analytical C18 column (PepSep, 1.9 μm^3^ beads, 75 µm ID) and separated over a 60 min gradient. The gradient started at 7% Buffer B (0.1% formic acid in 80% acetonitrile), increased to 35% over 40 min, then to 45% within 5 min, followed by a rapid ramp to 99% in 0.5 min and equilibration for 14.5 min.

Full MS spectra were acquired in profile mode using a Q Exactive HF-X Hybrid Quadrupole Orbitrap mass spectrometer operating in the scan range of 375–1400 m/z using 120 K resolving power with an AGC of 3 × 10^6^ and max IT of 60 ms followed by data independent analysis using 8 Th windows (39 loop counts) with 15 K resolving power with an AGC (automatic gain control) of 3 × 10^5^ and max IT of 22 ms and a normalized collision energy (NCE) of 26. Each biological replicate was analyzed in three technical replicates on the system.

#### Data analysis

Raw Orbitrap data were analyzed in DIA-NN v2.0 (Data-Independent Acquisition by Neural Networks^54^ through searching against the reviewed canonical *Homo sapiens* (20 757 proteins) database and the DIA-NN contaminant database in the library free mode of the software, allowing up to two tryptic missed cleavages. Protein quantification was performed using the QuantUMS (Quantification using an Uncertainty Minimization Solution) high-precision approach, with scoring parameters set to generic. A spectral library was created from the DIA runs and used to reanalyze them. DIA-NN default settings were used with oxidation of methionine residues and acetylation of the protein N-termini set as variable modifications and carbamidomethylation of cysteine residues as fixed modifications. N-terminal methionine excision was enabled. The match between runs (MBR) feature was used for all analyses, and the output (precursor) was filtered at 1% FDR. Finally, protein inference was performed on the level of genes using only proteotypic peptides.

The generated results were processed statistically and visualized in Perseus software (1.6.15.0).^55^ The QuantUMS/MaxLFQ protein group quantities (total of 4938) were uploaded and Log2 transformed. The dataset was filtered, so the proteins from the CRAPome were removed. Additionally, a sample with a low number of identified proteins was removed (less than 50% of the total number of proteins in the initial dataset), as well as the proteins present with less than 70% valid values in at least one group. The remaining missing values were imputed using the default values in Perseus. The statistical analysis was performed in Perseus using the Welch *t* test when comparing two groups and multiple group testing using ANOVA. The ANOVA significant proteins were subjected to principal component analysis (Fig. 4b). Enrichment analysis was performed using the online tools STRING^56^ and Metascape.^57^ Supplementary Table S2 lists the significantly deregulated proteins identified by ANOVA across multiple group comparisons: IR vs. naïve vs. treatments (Fig. 4c), naïve vs. treatments (Fig. 4d), and IR vs. treatments (Fig. 4e). The table also contains the significant pairs of groups of each deregulated protein. Additionally, it contains the significant deregulated proteins as shown in the volcano plot (Fig. 4f) as a result of the *t* test comparison of the IR vs Treatments.

### Measurement of formate concentration

T cells were isolated from frozen PBMCs and activated using Dynabeads Human T-Activator CD3/CD28 beads in RPMI-1640 media (Gibco, Cat. 61870044) supplemented with 10% (v/v) dialyzed FBS (Gibco, Cat. A3382001) and penicillin-streptomycin (100 U/ml and 100 μg/ml, respectively; Gibco, Cat. 15070063). Cells were seeded at 1 × 10^5^ per well (with at least 3 × 10^6^ T cells per donor in total) in 96-well round bottom plates and activated for 24 h. After 24 h, culture supernatants (secreted formate) and cell lysates (intracellular formate) were collected, and formate concentrations were measured according to the manufacturer’s instructions (Formate Assay Kit, Cell Biolabs, Cat. MET-5133). Briefly, the assay is based on the conversion of a colorimetric probe to a formazan product in the presence of formate and formate dehydrogenase (FDH), which is detected spectrophotometrically at 450 nm. For the formate detection of cell lysates (intracellular formate), formate levels were normalized to the number of seeded cells per donor. The assay plates were read using a HIDEX SENSE Multimode plate reader.

### Flow cytometry

For analysis of immune cells, single-cell suspensions from primary human pan T-cell cultures or mouse splenocytes, lymph nodes and blood samples were stained with antibodies against the targets described in Supplementary Table S3. Single-cell suspensions from mouse spleens and lymph nodes were generated by passing them through a 40 μm cell strainer (BD Falcon). For the spleens, the splenocytes were then subjected to erythrolysis (eBioscience 1X RBC Lysis Buffer, Cat. 00-4333-57) as well as for the blood samples prior to staining. For intracellular staining, MTHFD2, Ki67, Foxp3 and GATA3 cells were fixed and stained using the Foxp3 Staining Set (Cat. 00-5523-00, eBioscience) according to the manufacturer’s instructions. Data acquisition was performed on a NovoCyte Quanteon. Analysis was performed with FlowJo software. Apoptotic cells were always excluded from analysis using Zombie staining (Zombie Aqua Fixable Viability Kit, Cat. 423101) according to the manufacturer’s recommendations. Supplementary Table S3 includes details on the antibodies (fluorochrome, clone, vendor, catalog number, and concentration).

### Immunofluorescence and confocal microscopy

For the cell immunofluorescence experiments, freshly isolated pan T cells from peripheral blood were seeded onto poly-L-lysine-coated plates (Fisher Scientific, Cat. 10220772), fixed with 4% paraformaldehyde for 15 min at room temperature, and washed twice with DPBS. Cells were blocked and permeabilized with 1% bovine serum albumin (BSA) in DPBS containing 0.1% Triton X-100 (blocking buffer) for 30 min at room temperature. Cell-seeded slides were then incubated with primary antibodies diluted in blocking buffer for 1.5 h at room temperature, followed by three washes with DPBS containing 0.1% Triton X-100. Secondary antibody incubation was carried out for 45 min at room temperature in the dark. Finally, the cells were mounted with ProLong Diamond Antifade Mountant with DAPI (Thermo Fisher Scientific, Cat. P36966) and visualized using a Zeiss LSM 780 confocal imaging system and Nikon AX R. Quantification of foci per cell or fluorescence intensity per cell was conducted using a macro developed in Fiji software. Nuclear expression of NFATC1 was calculated in CellProfiler. The primary antibodies for immunofluorescence were against MTHFD2 (Cell Signaling, Cat. 41377S; 1/200), MTHFD1 (Atlas, Cat. HPA000704; 1/230), and NFATC1 (Invitrogen, Cat. MA3-024; 1/250), and the secondary antibodies were Alexa Fluor 647 anti-rabbit (Invitrogen, Cat. A-31573; 1/300), Alexa Fluor 488 anti-rabbit (Invitrogen, Cat. A-11008, 1/500) and Alexa Fluor 568 anti-mouse (Invitrogen, A10037, 1/500) (Supplementary Table S3). For the analyses, at least 100 cells per human subject (corresponding to a minimum of four different fields of the coverslip) were observed for each marker under confocal microscopy. Validation of MTHFD2 and MTHFD1 antibody specificity by isotype and secondary antibody control staining is shown in Supplementary Material.

### RNA isolation and quantitative PCR analysis (real-time RT-PCR)

Human cells were lysed in buffer RA1 (Macherey-Nagel), and RNA was extracted using a NucleoSpin RNA XS isolation kit according to the manufacturer’s instructions. First-strand complementary DNA synthesis was performed using the PrimeScript RT-PCR Kit (Takara, Cat. RR037A).

qPCR was carried out using iTaq Universal SYBR Green Supermix (Bio-Rad, Cat. 1725124). Relative expression of target genes was calculated by comparing them to the expression of the housekeeping gene *ACTB*. The primer sequences that were used for real-time reverse transcription qPCR (RT-qPCR) were as follows: *ACTB* forward, 5′-CCTGGCACCCAGCACAAT-3′; *ACTB* reverse, 5′-GGGCCGGACTCGTCATACT-3′; *MTHFD2* forward, 5′-GGGAAGAATGTGGTTGTGGC-3′; *MTHFD2* reverse, 5′-ATGACTGCTGCTCCTTCCTT-3′; *MTHFD1* forward, 5′-CCCTTAGGCGTACAAGGAATG-3′; *MTHFD1* reverse, 5′-AGGATGTGGATGGATTGACTAGC-3′; *IL2RA* forward, 5′-GAGACTTCCTGCCTCGTCACAA-3′; *IL2RA* reverse, 5′-GATCAGCAGGAAAACACAGCCG-3′; *IL2* forward, 5′-AGAACTCAAACCTCTGGAGGAAG-3′; *IL2* reverse, 5′-GCTGTCTCATCAGCATATTCACAC-3′; *NFKB1* forward, 5′- GCAGCACTACTTCTTGACCACC-3′; *NFKB1* reverse, 5′-TCTGCTCCTGAGCATTGACGTC-3′; *NFATC1* forward, 5′-CACCAAAGTCCTGGAGATCCCA-3′; *NFATC1* reverse, 5′-TTCTTCCTCCCGATGTCCGTCT-3′; *IL6* forward, 5′-AGACAGCCACTCACCTCTTCAG-3′; *IL6* reverse, 5′-TTCTGCCAGTGCCTCTTTGCTG-3′; *CCL5* forward, 5′-TGCCACTGGTGTAGAAATACTC-3′; *CCL5* reverse, 5′- GCTGTCATCCTCATTGCTACT-3′; *CXCL10* forward, 5′-GACATATTCTGAGCCTACAGCA-3′; *CXCL10* reverse, 5′-CAGTTCTAGAGAGAGGTACTCCT-3′; *CCL20* forward, 5′-CCATGTGCTGTACCAAGAGT-3′; *CCL20* reverse, 5′-TTAGGATGAAGAATACGGTCTGTG-3′; *IL1B* forward, 5′-CCACAGACCTTCCAGGAGAATG-3′; *IL1B* reverse, 5′-GTGCAGTTCAGTGATCGTACAGG-3′; *TNF* forward, 5’-TGCACTTTGGAGTGATCGG-3′; *TNF* reverse, 5′-TCAGCTTGAGGGTTTGCTAC-3’; *IFNG* forward, 5′-CGACAGTTCAGCCATCACTT-3’; *IFNG* reverse, 5′-GCAACAAAAAGAAACGAGATGAC-3′. Real-time PCR was performed using a QuantStudio™ 5 Real-Time PCR System.

### Measurement of cytokines

#### Legendplex

Secreted cytokines in RA ex vivo cell culture supernatants were measured with a LEGENDplex HU Essential Immune Response Panel (Cat. 740930) following the collection of supernatants 65 h post-culture of RA T cells treated with TH9169 (1 µM), MTX (1 µM) or vehicle (DMSO). Data acquisition was performed on a NovoCyte Quanteon. Analysis was performed with the LEGENDplex Data Analysis Software Suire from Qognit. Similarly, murine plasma cytokines were measured with an MU Inflam Panel (Cat. 740446) at the endpoint of the in vivo studies.

#### ELISA

ELISA for the detection of serum anti-GPI antibodies was performed. Briefly, 96-well flat-bottom ELISA plates (Maxisorb; Nunc) were coated with 10 μg/ml recombinant GPI (Sigma, Cat. P5381) in PBS at 4 °C overnight. The plates were blocked with BSA before the addition of serum (1:50 dilution). After washing, the cells were incubated with mouse HRP-conjugated IgG (Santa Cruz Biotechnology, Cat. sc-2005). TMB substrate (Medicago, Cat. 10-9405-250) was added after washing, and the plate was read by an ELISA reader at 450 nm.

### Chromatin immunoprecipitation (ChIP) assay

To investigate the molecular interactions of MTHFD2 in T cells ex vivo, ChIP assays were performed. Briefly, 2 × 10^5^ cells per well were seeded in a 96-well round-bottom plate in HPLM supplemented medium with or without CD3/CD28 T-Activator Dynabeads as previously described. Cells were collected 6 h after culture, and ChIP was performed with the Magna ChIP A/G Chromatin Immunoprecipitation Kit (Merck, Cat. 17-10085) following the manufacturer’s instructions. Immunoprecipitation was carried out using antibodies against NFATC1 (Invitrogen, Cat. MA3-024; 2 µl per reaction from 100 µl stock) and negative control IgG1 (Cell Signaling, Cat. 5415S). Enriched DNA fragments were analyzed by quantitative real-time PCR using primers targeting the *MTHFD2* promoter-proximal region. In all cases, data (*C*_t_ values) derived from the input sample were used for normalization by the ‘percent of input (%IP)’ method and presented as fold of change relative to control anti-IgG1 IPs. The sequences of the core consensus response element for NFATC1 were identified on the *MTHFD2* promoter-proximal sequence using TFmotifView,^58^ the Gene Transcription Regulation Database^59^ and EPD.^60^ Primer sequences are listed below: *MTHFD2* loc1-forward, 5′-GCACACAGTAGGCGCTCATA-3′; *MTHFD2* loc1-reverse, 5′-AGTCAAACTCCTGCCTGACG-3′; *MTHFD2* loc2-forward, 5′-CGTCAGGCAGGAGTTTGACT -3′; *MTHFD2* loc2-reverse, 5′-GCCCTGCTTTTGATTGGAGC-3′; *MTHFD2* loc3-forward, 5′-AGAGCCCTGCACCTTACAAC-3′; and *MTHFD2* loc3-reverse, 5′-GCCCCTTGTCAGGAACATGA-3′.

For ChIP experiments with Jurkat cells, 2.5 × 10^5^ cells per well were seeded in a 96-well round-bottom plate (4 wells per condition) in RPMI1640 supplemented medium in the presence of CD3/CD28 T-Activator Dynabeads as previously described and TH9619 (1 µM) for 6 h. Hypoxanthine (10 µM) (Sigma, Cat. H9636) was added to the Jurkat cell culture 4 days prior to the addition of Dynabeads and TH9619.

### NFATC1 knockdown assay

To establish NFATc1 knockdown cells, NFATc1 siRNA was purchased from Life Technologies (Silencer™ Select Pre-Designed siRNA, 4392420-s9472). Pan T cells were isolated from PBMCs with an EasySep™ Human T-Cell Isolation Kit and stimulated with Dynabeads Human T-Activator CD3/CD28. siRNA (250 nM) was transfected using INTERFERin® transfection reagent (Polyplus, 101000028) following the manufacturer’s instructions. A nontargeting control siRNA (All-Stars Negative Control, Qiagen, 1027280) was used at the same concentration. Transfections were performed twice to enhance knockdown efficiency. After transfection, the cells were incubated for the indicated time points before further experiments.

### Western blotting

Cells were washed with phosphate-buffered saline (PBS) and resuspended in 50 µl of RIPA buffer (Thermo Fisher Scientific) supplemented with cOmplete™ protease inhibitor cocktail (Roche) and Halt™ phosphatase inhibitor cocktail (Thermo Fisher Scientific). Samples were lysed by sonification and centrifuged to obtain whole-cell extracts. Cleared lysates were denatured and reduced using NuPAGE™ LDS sample buffer and NuPAGE™ sample reducing agent (Thermo Fisher Scientific). Proteins were resolved on 4-15% PROTEAN® TGX™ precast gels (Bio-Rad) and subsequently transferred onto PVDF membranes (Bio-Rad). Membranes were blocked with 5% (w/v) nonfat dry milk in TBST (Tris-buffered saline with 0.1% Tween-20), followed by incubation with primary antibodies overnight at 4 °C. Actin and histone H3 were used as loading controls. After washing, the membranes were incubated with appropriate secondary antibodies for 1 h at room temperature. Protein bands were visualized using an Odyssey® Fc Imaging System (LI-COR Biosciences) and quantified using ImageJ software. Uncropped Western blot images are shown in Supplementary Material.

### CRISPR/Cas9-mediated MTHFD2 knockout in Jurkat cells

Jurkat cells were washed with PBS and resuspended in Buffer R from the Neon™ Transfection System 10 µl kit (Thermo Fisher Scientific). Electroporation was performed using the Neon™ Transfection System according to the manufacturer’s instructions. Synthetic guide RNAs targeting MTHFD2 were obtained from Synthego, and TrueCut™ Cas9 Protein v2 was purchased from Thermo Fisher Scientific. Following electroporation, cells were cultured for 96 h and then seeded at a single-cell density in 96-well plates. Individual clones were screened by PCR and confirmed by Sanger sequencing and western blot analysis for complete MTHFD2 knockout.

### Calcineurin inhibitor treatment

Jurkat cells were cultured in RPMI-1640 medium supplemented with 10% dialyzed fetal bovine serum (FBS) and 10 µM hypoxanthine for four days prior to the experiment. Cells were stimulated with CD3/CD28 activation beads (Gibco) and treated with 1 µM TH9619, tacrolimus (FK506; MedChemExpress), or Cyclosporin A (CycA; MedChemExpress) for 6 or 24 h. Cells were then harvested for quantitative PCR and immunofluorescence microscopy analyses.

### Data analysis and statistics

Statistical analyses were performed using GraphPad Prism v10. Data are presented as volcano plots showing all data points or as the mean ± SEM unless otherwise stated. SuperPlots^61^ were used where specified to visualize both within-donor and between-donor variation. Statistical tests were selected according to data distribution and experimental design: two-tailed Student’s *t* test or Mann-Whitney *U* test for pairwise comparisons and one-way or two-way ANOVA or Kruskal-Wallis tests for multiple-group analyses. Paired, repeated-measures, or unpaired analyses were applied as appropriate. For all tests, *P* < 0.05 was considered significant. Statistical methods for all-omics datasets are detailed in their respective Methods subsections. Investigators were not blinded to sample identity. All compared samples were processed in parallel under identical conditions.

## Supplementary information

Supplementary Materials

Table S1

Table S2

Table S3

## Data Availability

The mass spectrometry proteomics data have been deposited to the PRIDE partner repository and are publicly accessible with the dataset identifier PXD061849. The detailed analyses of Fig. 4 (proteomics) can be found in Supplementary Table S2. Uncropped Western blot images can be found in Supplementary Material. Publicly available data that support the findings of this study were obtained from the NCBI Gene Expression Omnibus (GEO) under the accession numbers GSE118829 and GSE93777 and the European Genome-phenome Archive (EGA) under accession number EGAS00001005241. All other data are available in the article and its Supplementary files or from the corresponding author upon reasonable request.
