# Supplementary material for: Targeting NFATc1-regulated MTHFD2 one-carbon metabolism to suppress sustained T-cell-mediated inflammation in rheumatoid arthritis
Source: Signal Transduct Target Ther. 2026 Jun 10;11:226. doi: 10.1038/s41392-026-02752-y (PMC13254404; doi:10.1038/s41392-026-02752-y)
Supplement: Supplementary file 1 — Supplementary Materials [file 41392_2026_2752_MOESM1_ESM.pdf]

# Supplementary Materials for

## Targeting NFATc1-regulated MTHFD2 one-carbon metabolism to suppress sustained T cell-mediated inflammation in rheumatoid arthritis

Theodora Manolakou[1]*, Jianyu Shen[1]#, Sanjaykumar Boddul[2]#, Martina Samiotaki[3], Michail Angelos Panagias[1], George Sentis[4], Tarcília Aparecida Silva[5], Alexandra Argyriou[2], Dionysis Nikolopoulos[2], Kumar Sanjiv[1], Karine Chemin[2], Fredrik Wermeling[2], Martin Henriksson[1], Ana Slipicevic[1,6], Per-Johan Jakobsson[2], Katerina Chatzidionysiou[2] and Thomas Helleday[1,7]*

Affiliations:

[1]Science for Life Laboratory, Department of Oncology-Pathology, Karolinska Institutet, Solna, Sweden.

[2]Center for Molecular Medicine, Division of Rheumatology, Department of Medicine, Solna, Karolinska University Hospital, Karolinska Institutet, Stockholm, Sweden.

[3]Institute for Bioinnovation, Biomedical Sciences Research Centre Alexander Fleming, Vari, Athens, 166 72, Greece.

[4]Clinical, Experimental Surgery & Translational Research, Biomedical Research Foundation of the Academy of Athens, Athens, Greece.

[5]Department of Oral Surgery, Pathology and Clinical Dentistry, School of Dentistry, Federal University of Minas Gerais, Belo Horizonte, Minas Gerais, Brazil.

[6]One-carbon Therapeutics AB, Stockholm, Sweden.

[7]Department of Oncology and Metabolism, Medical School, S10 2RX, Sheffield, UK.

#Equal contribution

*Corresponding

Correspondence to: theodora.manolakou@ki.se, thomas.helleday@scilifelab.se

**This PDF file includes:**

- Supplementary Figures 1 to 18 (and captions)
- Validation of MTHFD2 and MTHFD1 antibody specificity by isotype and secondary antibody control staining
- Uncropped Western blot images

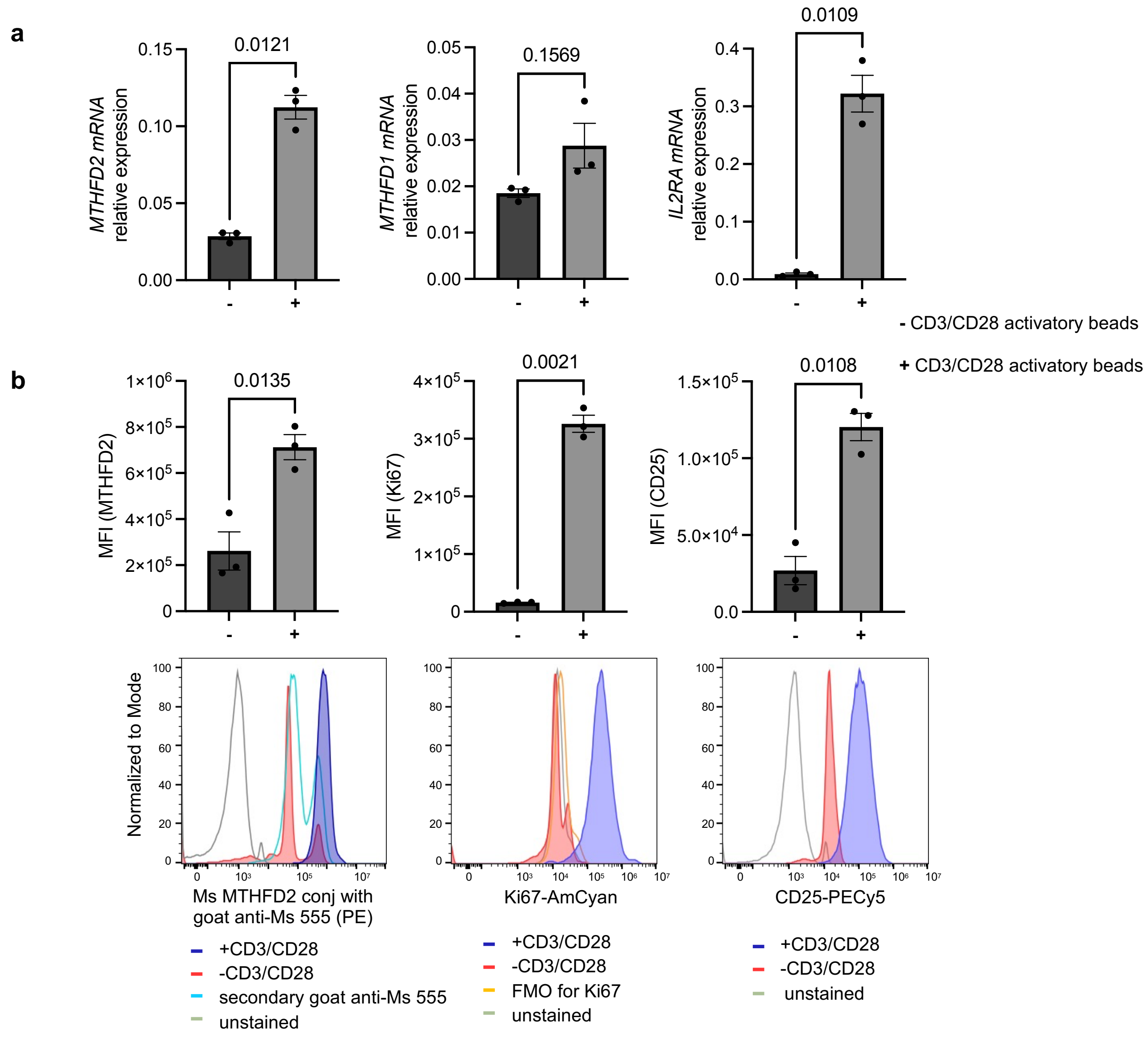


**Supplementary Figure 1.**

***Ex vivo* activation of T cells increases MTHFD2 expression.** Pan T cells were isolated from the peripheral blood of healthy donors (n = 3) using a magnetic bead-based approach and cultured in the presence or absence of CD3/CD28 stimulatory beads. **(a)** Relative expression levels of *MTHFD2, MTHFD1* and *IL2RA* mRNA measured with real-time RT-qPCR (normalized to *ACTB*) following culture for 24 hours. **(b)** Assessment of MTHFD2 protein expression, proliferation (Ki67) and activation (CD25) as assessed by flow cytometry following culture for 72 hours. The analyzed cells were zombie-negative (viable cells). Controls have been used as indicated on the relevant histograms. Statistical significance was obtained by paired *t* test. N = 3 per group. Data are represented as mean ± SEM. *P* values are indicated in the respective graph. MFI geometric mean fluorescent intensity

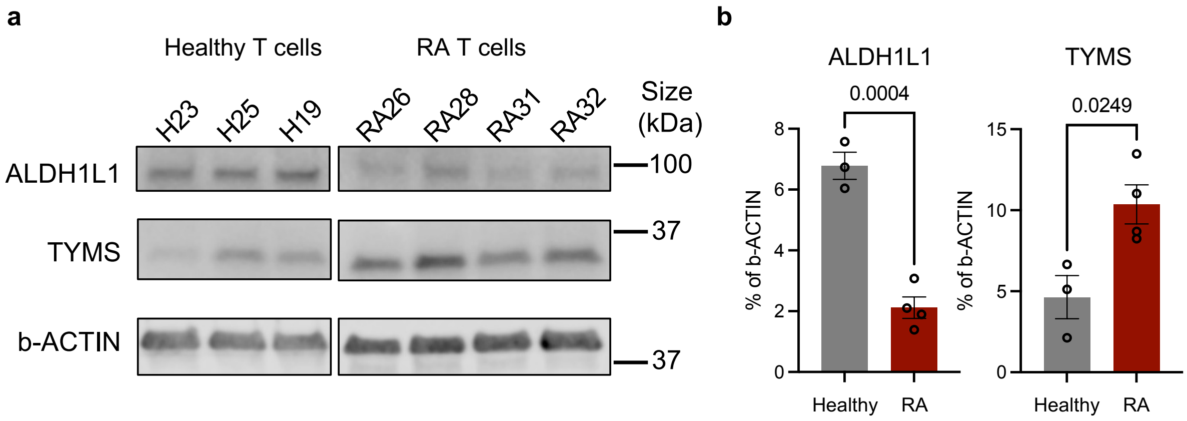


**Supplementary Figure 2.**

**Coordinated regulation of one-carbon metabolism enzymes in RA T cells. (a)** Western blot analysis of one-carbon metabolism-related proteins in pan T cells from healthy donors (n = 3) and treatment-naïve rheumatoid arthritis (RA) patients (n = 4). Pan T cells were isolated directly from PBMCs and snap-frozen prior to analysis. **(b)** Quantification of ALDH1L1 and TYMS protein levels normalized to β-ACTIN. Data are represented as mean ± SEM. Statistical significance was determined using an unpaired *t* test. *P* values < 0.05 are indicated in the respective graphs.

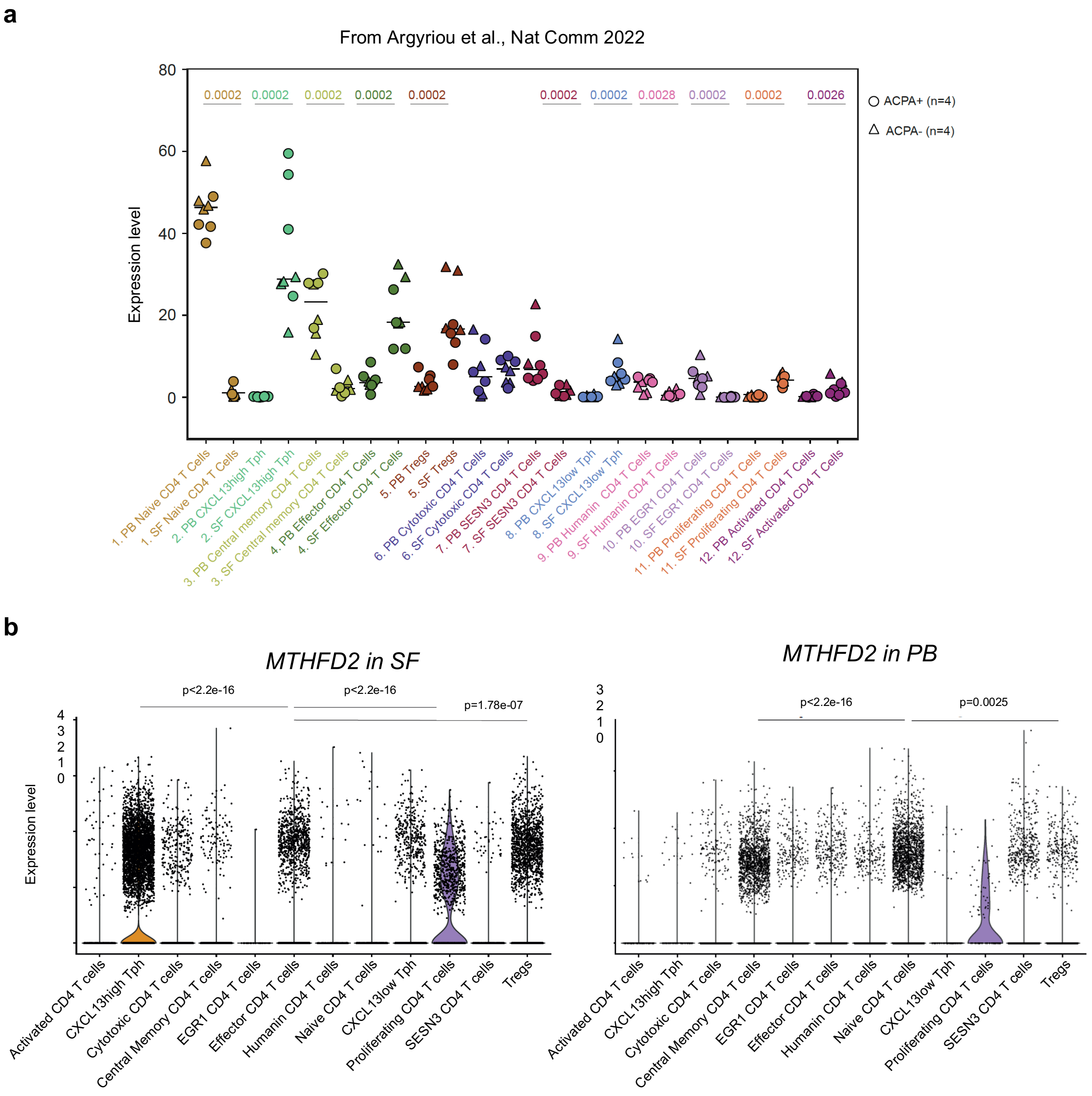


**Supplementary Figure 3.**

**Abundance of T-cell subsets in synovial fluid and peripheral blood of RA patients. (a)** Frequencies of CD4$^+$ T-cell clusters in peripheral blood (PB) and synovial fluid (SF) from n = 8 RA patients (4 ACPA$^+$, 4 ACPA$^-$), generated at *Argyriou A. et al., 2022*. Circle indicates ACPA$^+$ RA patients, triangle indicates ACPA$^-$ patients. Line represents median, two-tailed Mann-Whitney *U* test. **(b)** Violin plots showing the expression of *MTHFD2* in the different CD4$^+$ T-cell subsets in SF (left panel) and PB (right panel). Statistical comparison performed using Mann-Whitney *U* test to compare the distribution on the gene expression between selected clusters. RA rheumatoid arthritis, ACPA anti-citrullinated protein antibodies

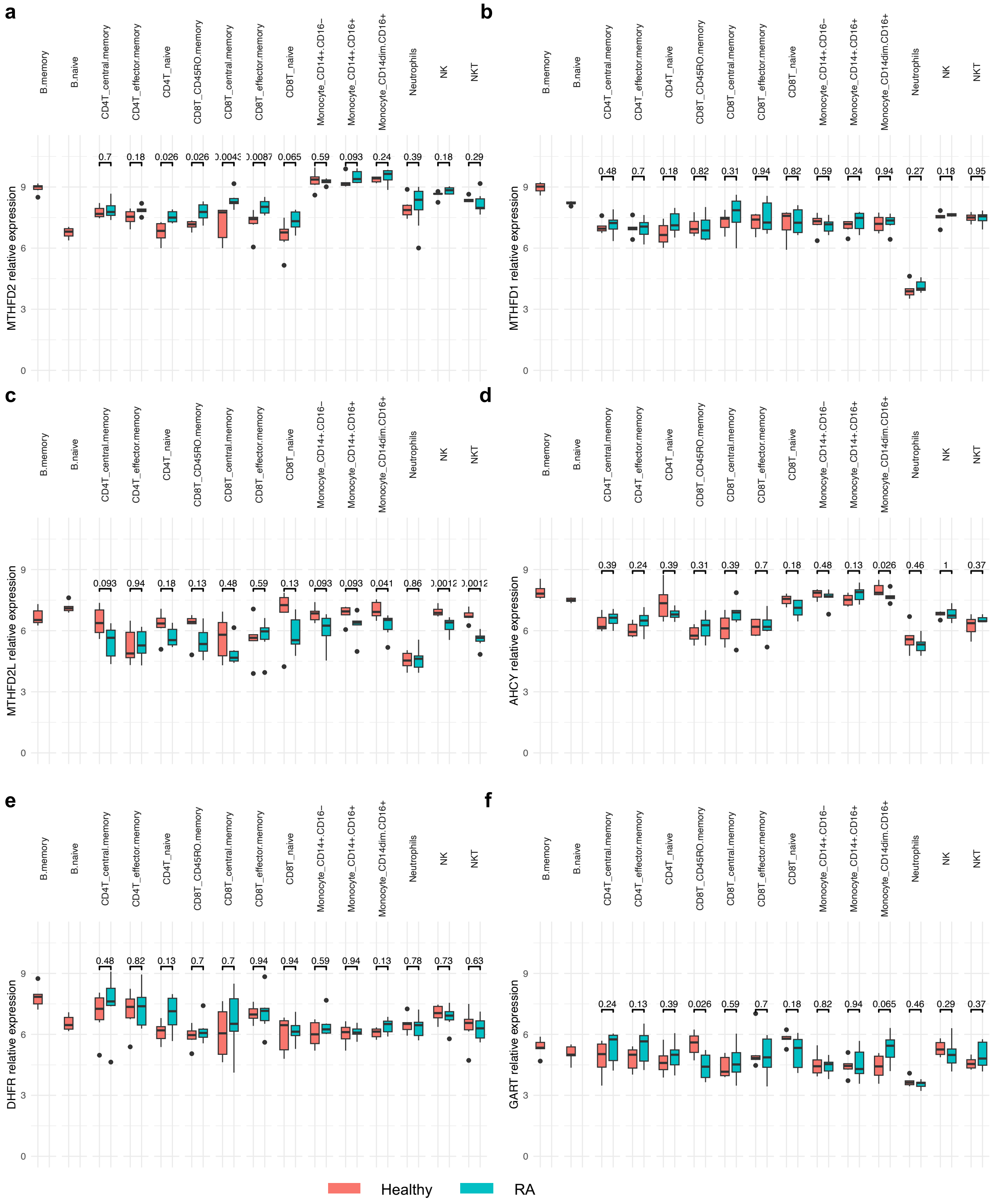


**Supplementary Figure 4.**

**Expression of one-carbon metabolism genes across immune cell types in RA.** Expression levels of (a) *MTHFD2*, (b) *MTHFD1*, (c) *MTHFD2L*, (d) *AHCY*, (e) *DHFR* and (f) *GART* in immune cell types isolated from peripheral blood (GSE93777 dataset). Color of the boxplots

represent sample phenotype with turquoise for rheumatoid arthritis (RA) and orange for healthy controls. Wilcoxon test was performed for statistical significance, with calculated $p$ values shown.

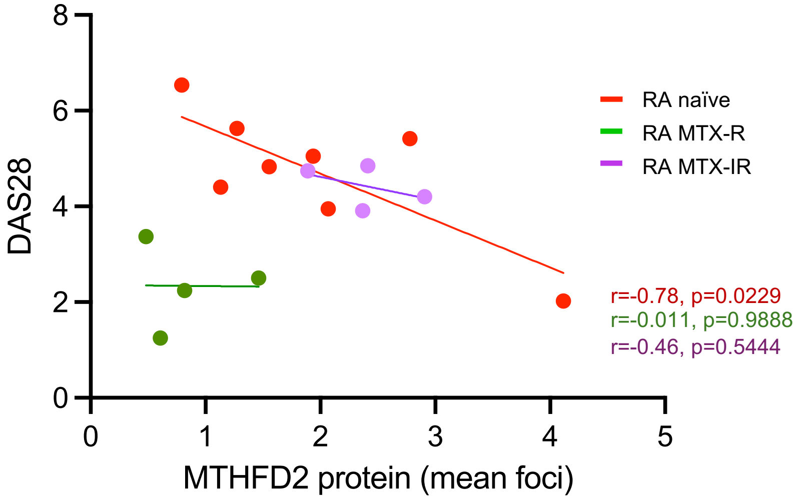


**Supplementary Figure 5.**
**Correlation between MTHFD2 protein levels in peripheral T cells and disease activity (DAS28) in RA.** Overlay of simple linear regression plots showing the relationship between MTHFD2 protein levels in peripheral T cells and DAS28 in treatment-naïve (n = 8, red), MTX-responder (MTX-R, n = 4, green), and MTX-inadequate responder (MTX-IR, n = 4, purple) RA patients. Lines represent simple linear regression fits; $r$ and $p$ values were calculated using Spearman's correlation. RA rheumatoid arthritis, MTX methotrexate, DAS28 disease activity score-28

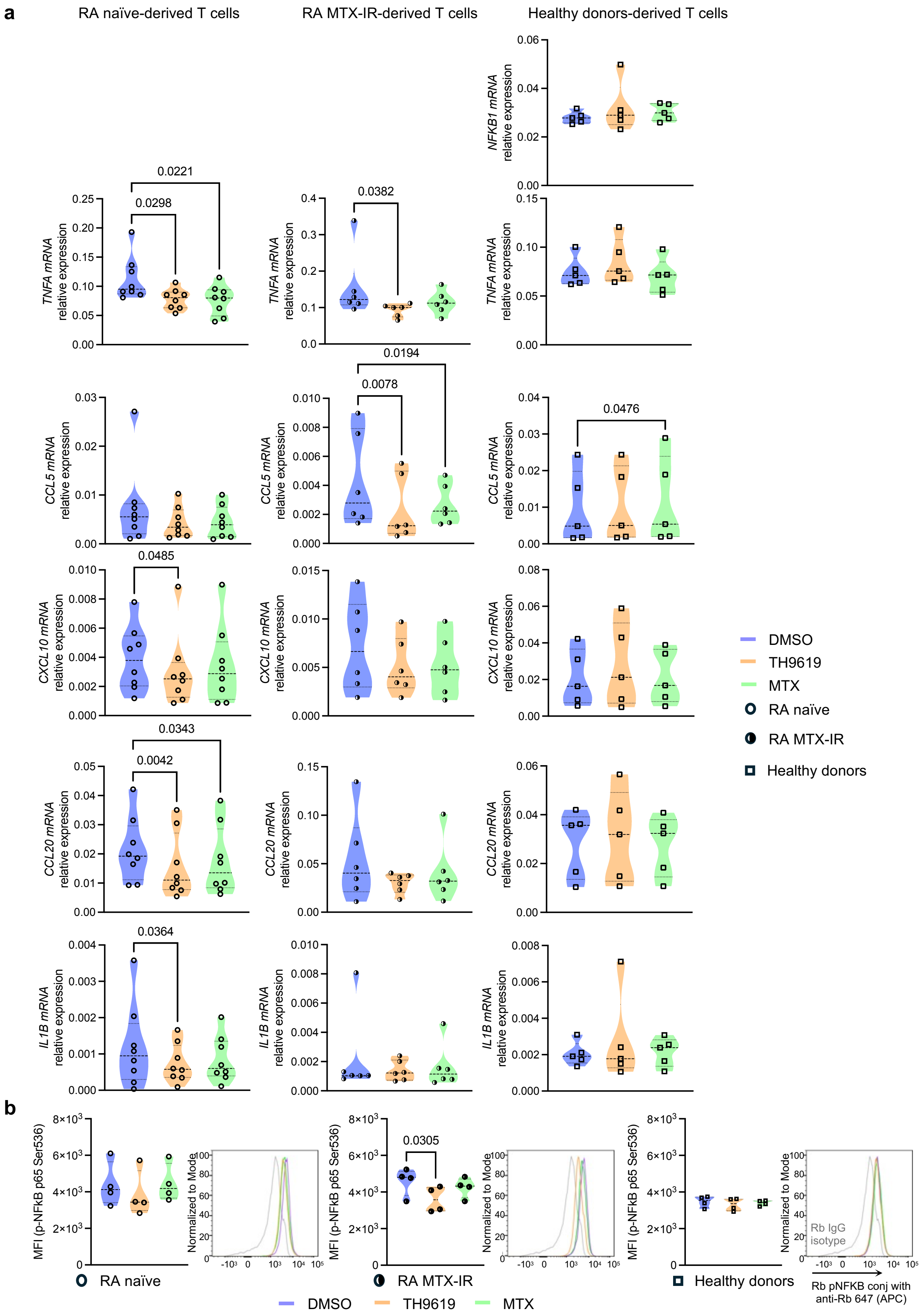
a
RA naïve-derived T cells
RA MTX-IR-derived T cells
Healthy donors-derived T cells
NFKB1 mRNA relative expression
TNFA mRNA relative expression
CCL5 mRNA relative expression
CXCL10 mRNA relative expression
CCL20 mRNA relative expression
IL1B mRNA relative expression
0.0221
0.0298
0.0382
0.0194
0.0078
0.0476
0.0485
0.0343
0.0042
0.0364
DMSO
TH9619
MTX
RA naïve
RA MTX-IR
Healthy donors
b
MFI (p-NFkB p65 Ser536)
Normalized to Mode
0.0305
Rb IgG isotype
Rb pNFKB conj with anti-Rb 647 (APC)
RA naïve
RA MTX-IR
Healthy donors
DMSO
TH9619
MTX

**Supplementary Figure 6.**

**TH9619 treatment alters NF-κB signaling in RA but not in healthy T cells. (a)** Pan T cells were isolated from the peripheral blood of RA patients that are treatment-naïve (n = 8), MTX-inadequate responders (MTX-IR, n = 6, posttreatment) and healthy donors (n = 5) using a magnetic bead-based approach and cultured with CD3/CD28 stimulatory beads in the presence or absence of MTHFD1/2i TH9619 (1 µM) or MTX (1 µM) for 24 hours. DMSO was used as vehicle. Relative mRNA expression levels of *NFKB1* target genes (e.g., *TNF, CCL5, CXCL10, CCL20,* and *IL1B*) measured with real-time RT-qPCR (normalized to *ACTB*) is shown. **(b)** Flow cytometric analysis of the geometric mean fluorescence intensity (MFI) of phosphorylated NF-κB p65 (Ser536) in pan T cells after 5 hours of stimulation with CD3/CD28 beads in the presence or absence of MTHFD1/2i TH9619 (1 µM) or MTX (1 µM). DMSO was used as vehicle. Pan T cells were isolated from PBMCs of treatment-naïve RA patients (n = 4), MTX-inadequate responders (MTX-IR, n = 4), and healthy donors (n = 4). All analyses were performed on zombie-negative (viable) cells. For all cases, statistical significance was obtained by one-way repeated measures ANOVA test followed by uncorrected Fisher's least significance difference (LSD) test. Data are represented as violin plots showing all data points. *P* values < 0.05 are indicated in the respective graph. RA rheumatoid arthritis, MTX methotrexate, IR inadequate responder

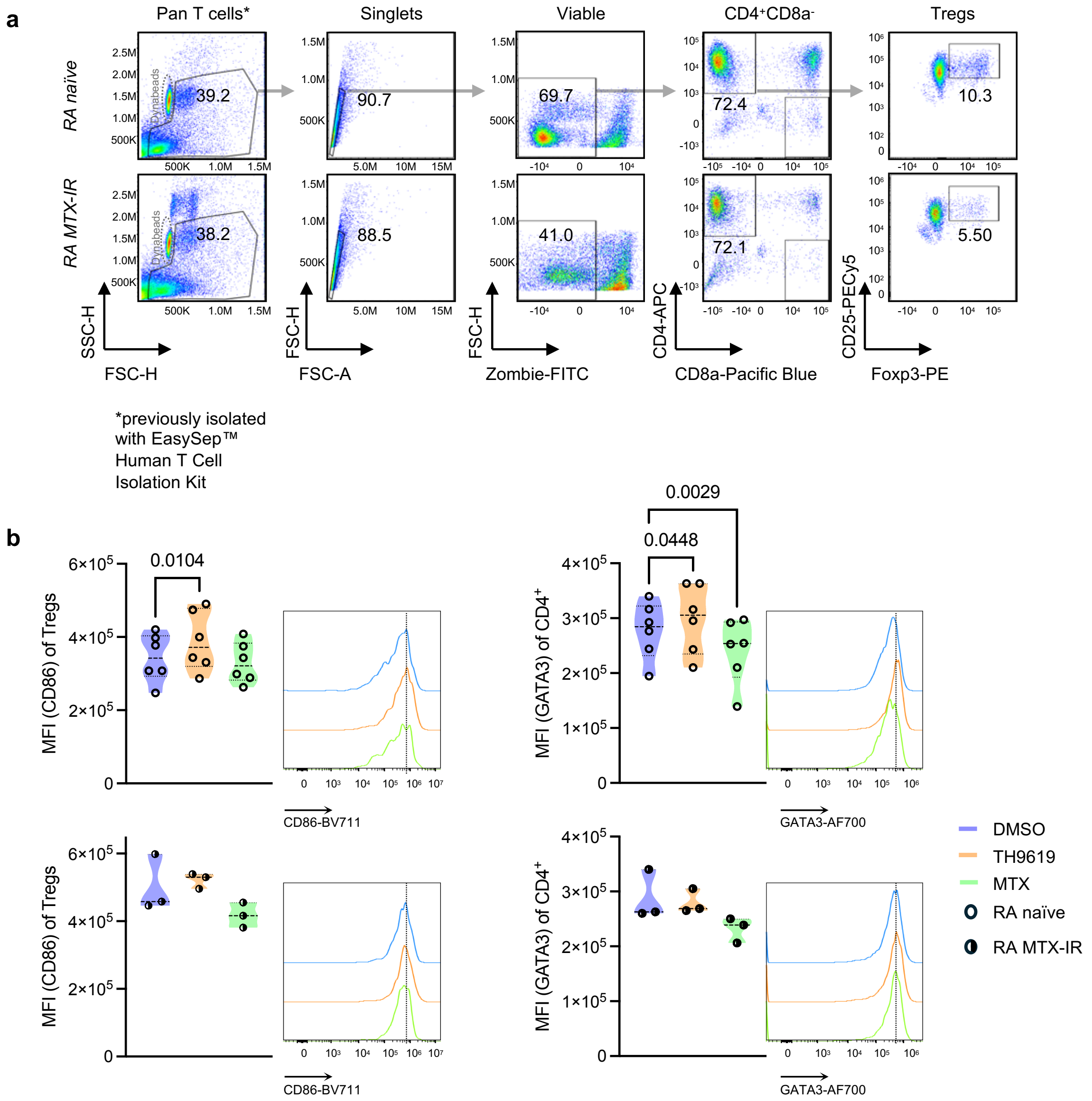


**Supplementary Figure 7.**

**TH9619 upregulates CD86 in Tregs and GATA3 in treatment-naïve RA T cells.** Pan T cells were isolated from the peripheral blood of RA patients that are treatment-naïve and MTX-inadequate responders (MTX-IR, posttreatment) using a magnetic bead-based approach and cultured with CD3/CD28 stimulatory beads in the presence or absence of MTHFD1/2i TH9619 (1 μM) or MTX (1 μM) for 24 hours. DMSO was used as vehicle. **(a)** Representative flow cytometry gating strategy of human samples. **(b)** Assessment of activation of T regulatory cell frequency (Tregs: $CD4^{+}CD25^{+}Foxp3^{+}$) and T helper cells ($CD4^{+}$) with flow cytometry with CD86 and GATA3 staining following culture of cells with the indicated compounds. The cells analyzed were zombie-negative (viable cells). Statistical significance was obtained by one-way repeated measures ANOVA test followed by uncorrected Fisher's least significance difference (LSD) test. Data are represented as violin plots showing all data points. *P* values < 0.05 are indicated in the

respective graph. RA rheumatoid arthritis, MTX methotrexate, IR inadequate responder, MFI geometric mean fluorescent intensity

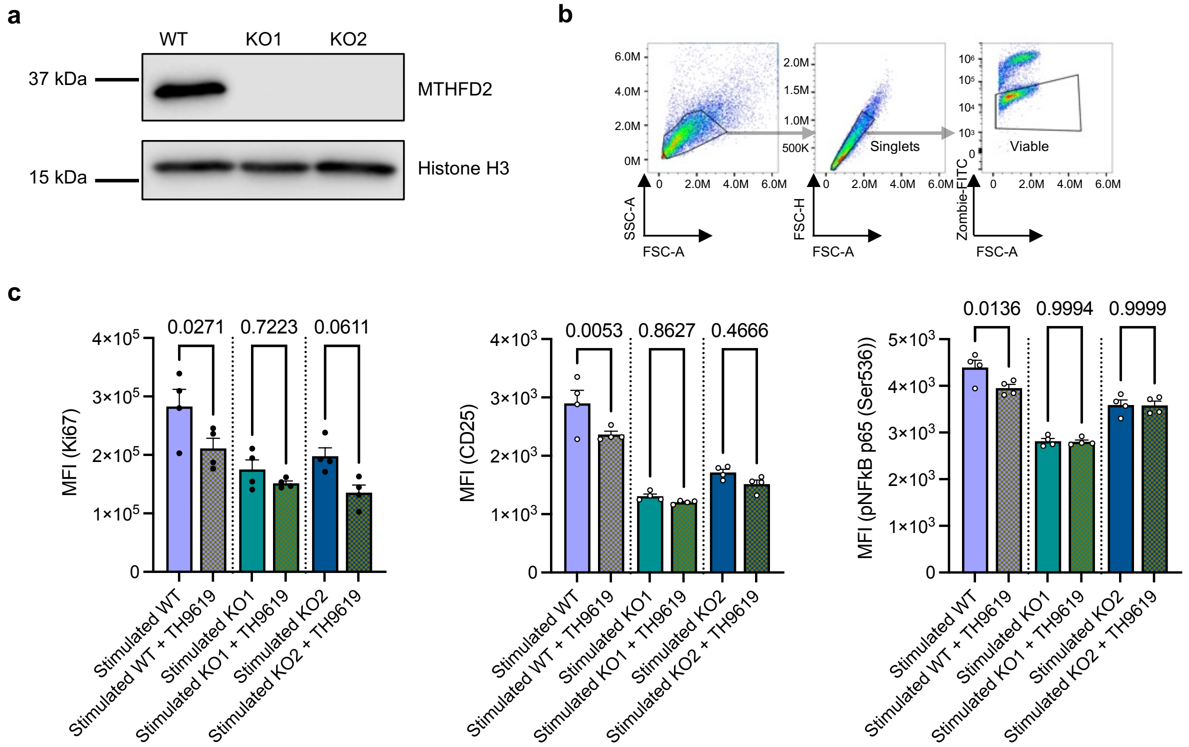


**Supplementary Figure 8.**

**MTHFD2 knockout abrogates TH9619 effects on T-cell proliferation and activation. (a)** Western blotting validation for MTHFD2 in wild type Jurkat cells (WT) and MTHFD2$^{-/-}$ Jurkat cells (KO1 and KO2). **(b)** Flow cytometry gating strategy. **(c)** Flow cytometric analysis of geometric mean fluorescence intensity (MFI) of Ki67 (left), CD25 (middle) and p-NF-κB p65 (Ser536) (right) markers within Jurkat cells. Cells were cultured with CD3/CD28 stimulatory beads and the indicated compounds for 24 hours. All analyses were performed in zombie-negative cells (viable cells). Data are represented as mean ± SEM. Statistical significance was obtained by one-way ANOVA. *P* values are indicated in the respective graph.

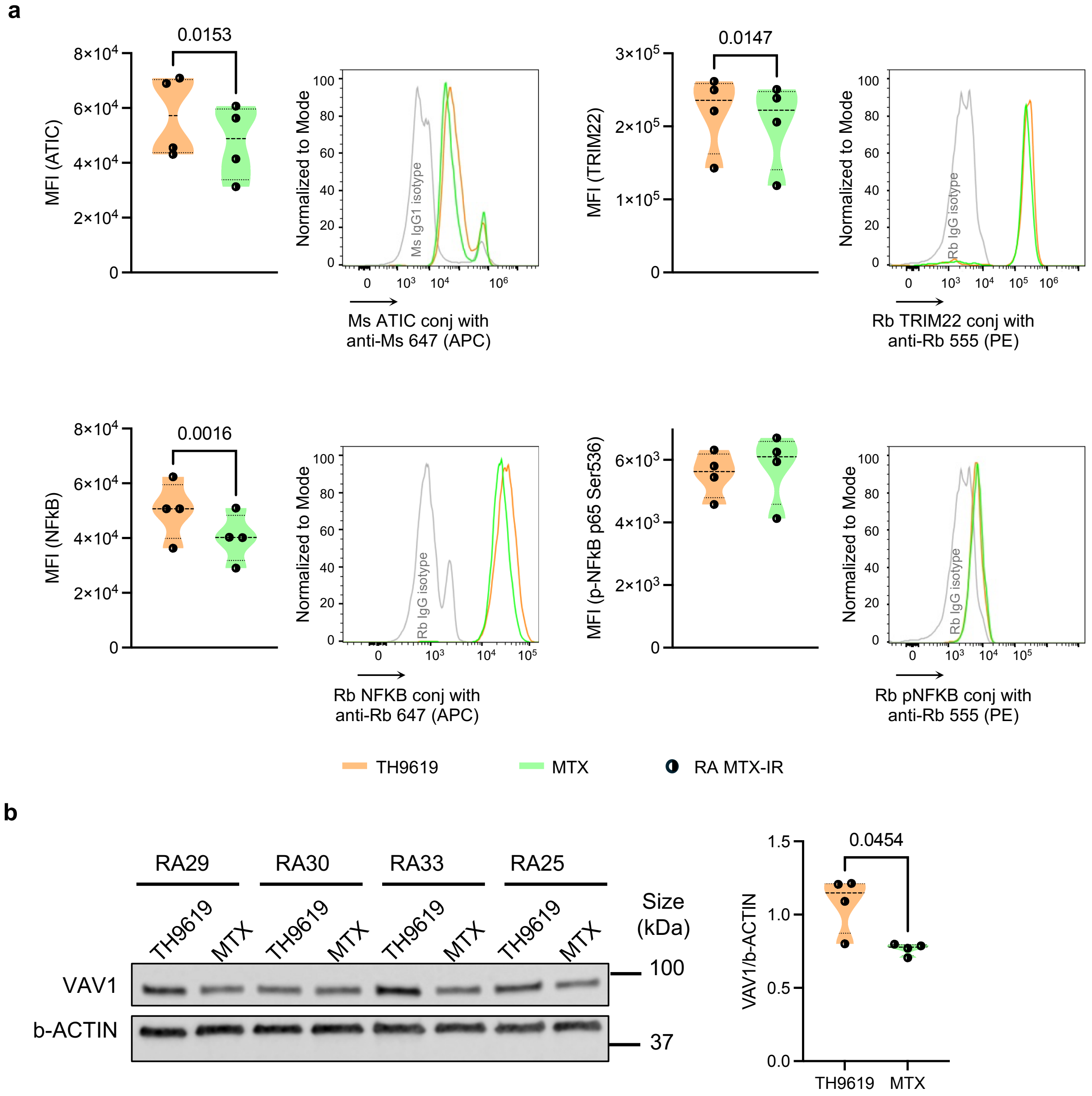


**Supplementary Figure 9.**

**TH9619 treatment increases ATIC, TRIM22, and NF-κB protein levels compared with MTX in MTX-IR RA T cells. (a)** Flow cytometric analysis of the geometric mean fluorescence intensity (MFI) of ATIC (top left), TRIM22 (top right), NF-κB p65 (bottom left), and phosphorylated NF-κB p65 (Ser536) (bottom right) in pan T cells after 24 hours of stimulation with CD3/CD28 beads in the presence of MTHFD1/2i TH9619 (1 μM) or MTX (1 μM). Pan T cells were isolated from PBMCs of MTX-inadequate responders (MTX-IR, n = 4). All analyses were performed on zombie-negative (viable) cells. **(b)** Western blot analysis of VAV1 protein in pan T cells from MTX-IR RA patients (n = 4) following treatment as in (a) and quantification of VAV1 protein (normalized β-ACTIN). For all cases, statistical significance was determined using a paired *t* test. Data are

represented as violin plots showing all data points. *P* values < 0.05 are indicated in the respective graphs. RA rheumatoid arthritis, MTX methotrexate, IR inadequate responder

- *Representative gating strategy (lymph nodes)*

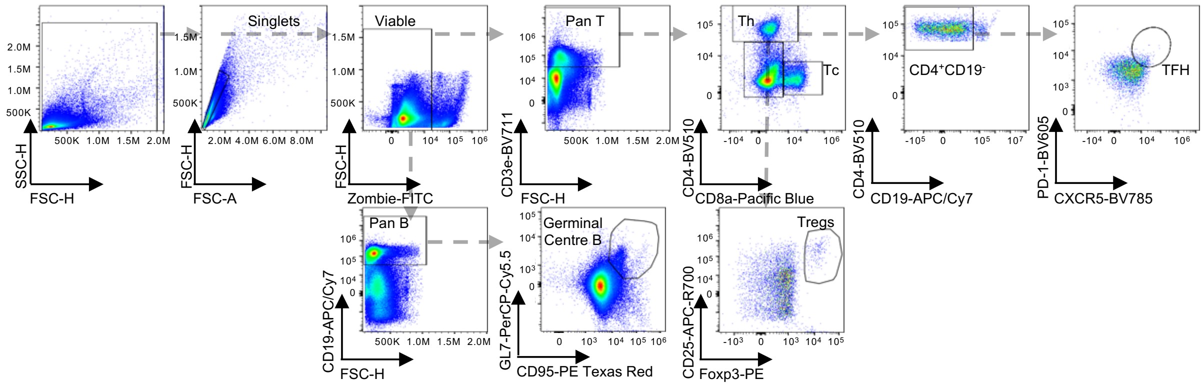


- *Representative gating strategy (spleen)*

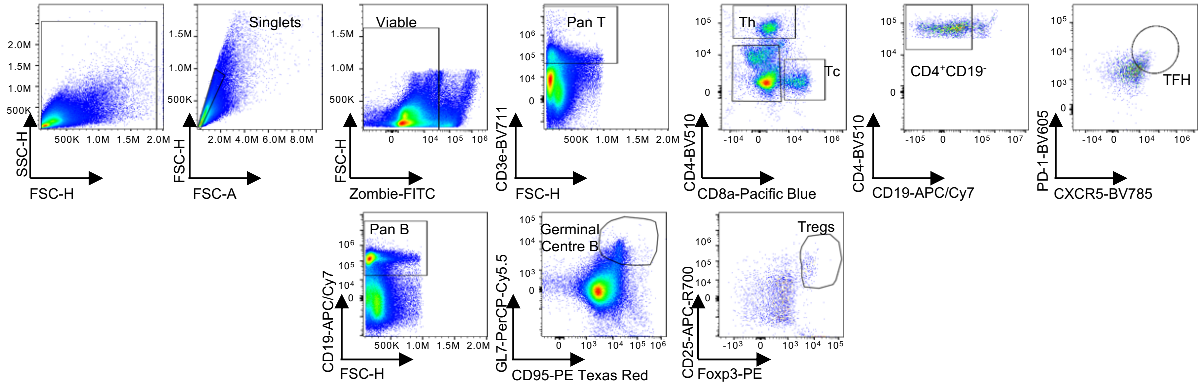


- *Representative gating strategy (blood)*

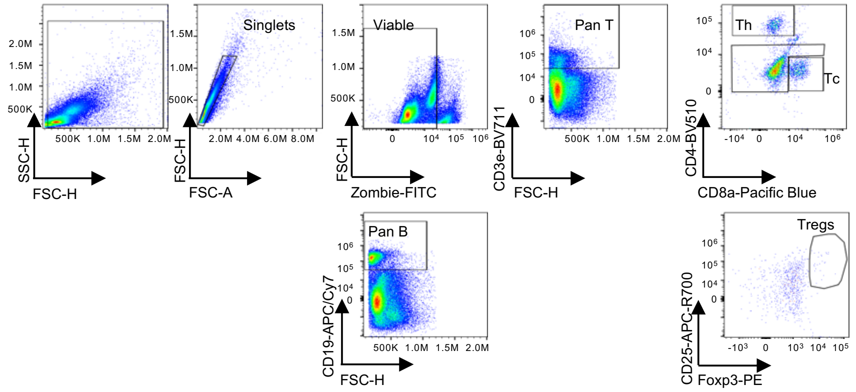


**Supplementary Figure 10.**
**Gating strategy of adaptive immune cells in murine inflammatory arthritis.** Flow cytometry gating strategy of $CD3^+$ pan T cells, $CD4^+$ T cells, $CD8\alpha^+$ T cells, $PD1^+CXCR5^+$ T follicular helper

cells (Tfh), CD25$^+$Foxp3$^+$ Tregs, CD19$^+$ pan B cells and GL7$^+$CD95$^+$ germinal center B cells (GC) in inguinal lymph node-, spleen- and blood- derived cells of the mice used in the *in vivo* studies.

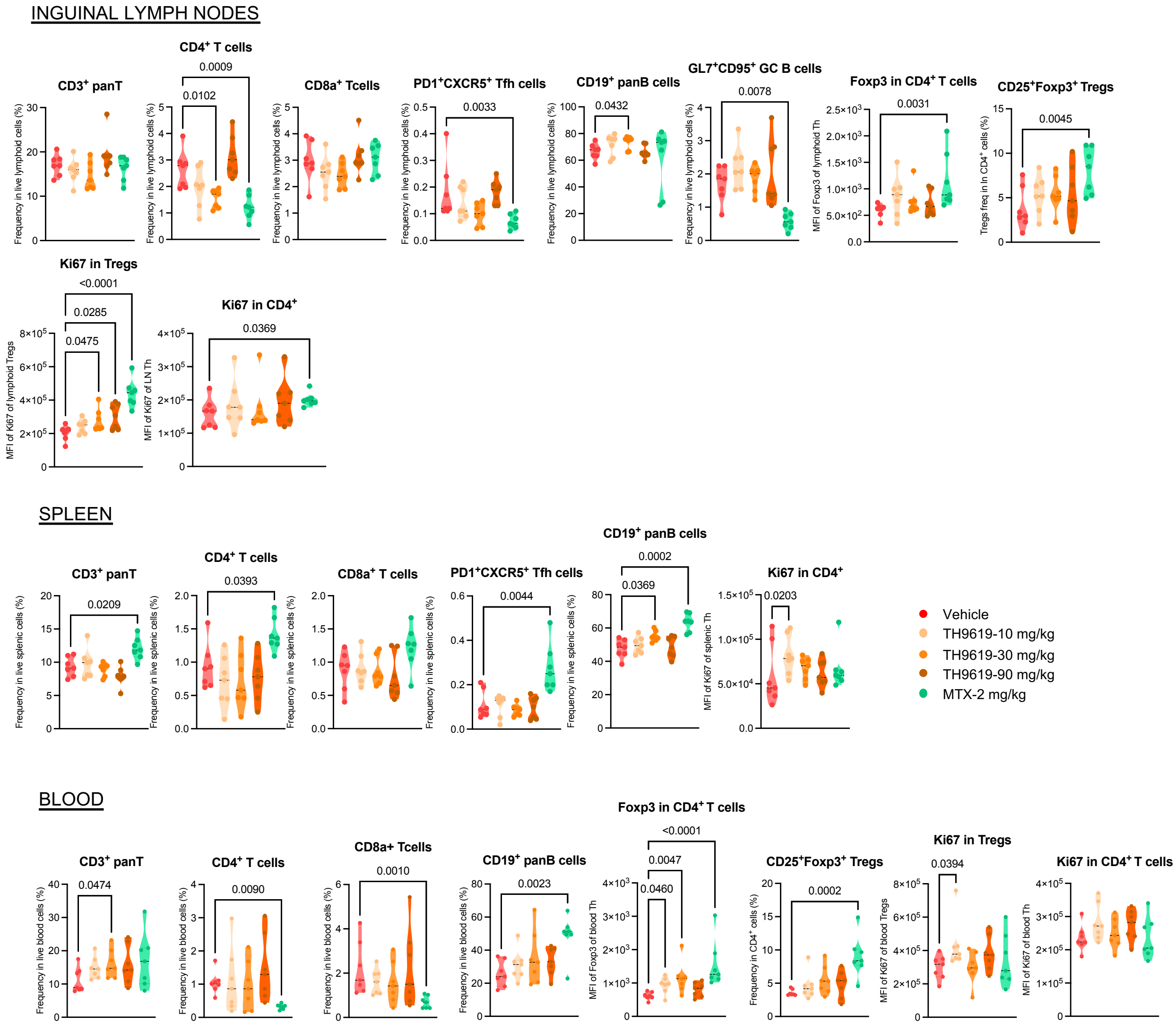


**Supplementary Figure 11.**

**Immunophenotyping of adaptive immune cells upon TH9619 treatment in murine inflammatory arthritis.** Immune profiling of the inguinal lymph node-, spleen- and blood-derived cells of the mice used in the *in vivo* studies, collected at the end of the experiment (day 11). Immunophenotyping was performed in zombie-negative cells (viable cells). Immune populations not shown in this figure are either included in the main figure or were not detectable in the respective tissues. N = 7 mice per group. Statistical significance was obtained by Kruskal-Wallis test followed by Dunn's uncorrected test. Data are represented as violin plots showing all data points. $P$ values < 0.05 are indicated in the respective graph. MFI geometric mean fluorescent intensity

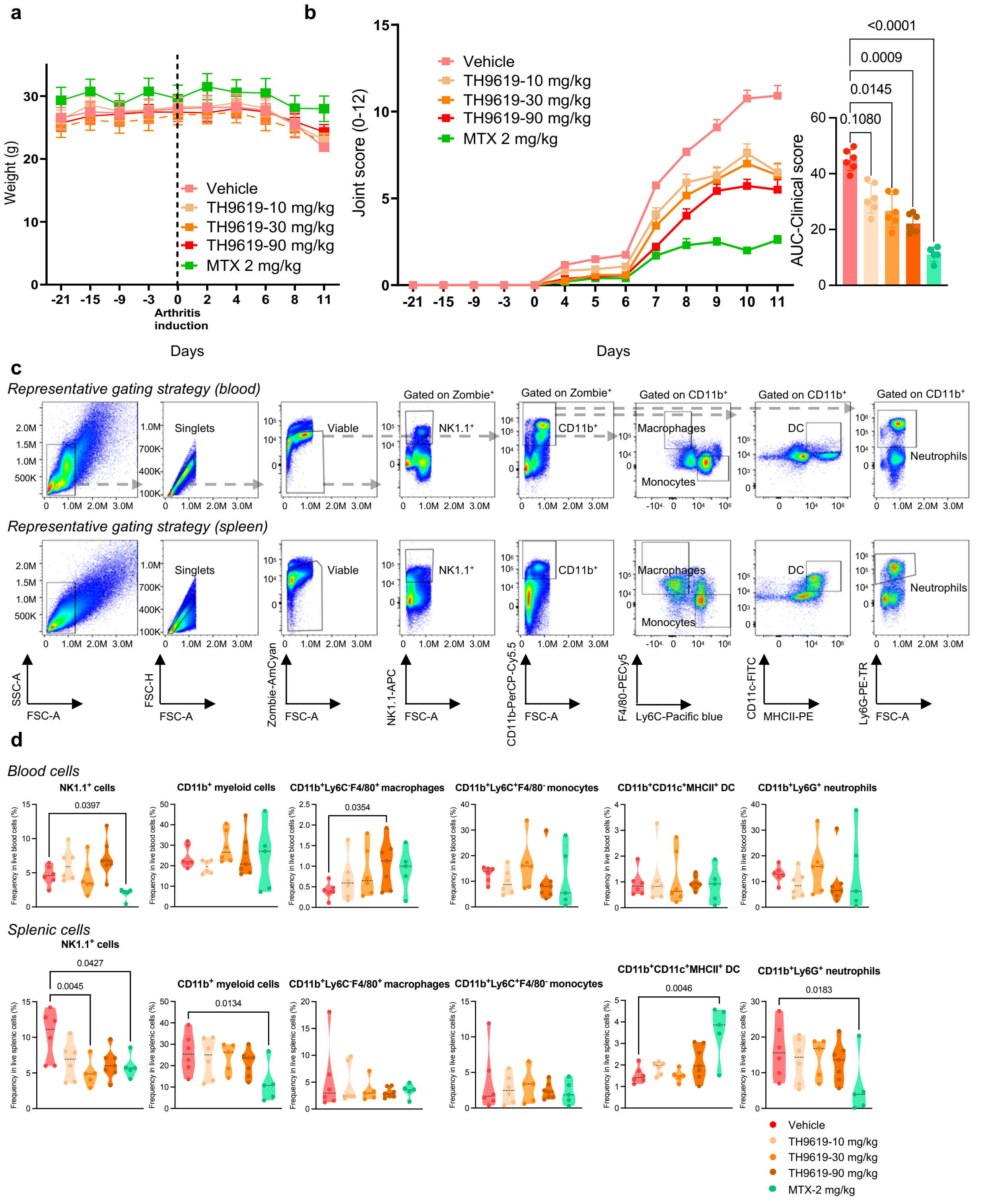


**Supplementary Figure 12.**

**Immunophenotyping of innate immune cells upon TH9619 treatment in murine inflammatory arthritis. (a)** Time course of daily body weight measurements in mice from the start of folate-depleted diet (21 days before inducing arthritis) to the experimental endpoint (day

11). **(b)** Left: Daily assessment of joint score based on clinical criteria. Right: Quantification of total joint score based on the area under curve of the graph on the left. **(c)** Gating strategy of NK1.1$^{+}$ cells, CD11b$^{+}$ myeloid cells, CD11b$^{+}$Ly6C$^{-}$F4/80$^{+}$ macrophages, CD11b$^{+}$Ly6C$^{+}$F4/80$^{-}$ monocytes, CD11b$^{+}$CD11c$^{+}$MHCII$^{+}$ dendritic cells (DC) and CD11b$^{+}$Ly6G$^{+}$ neutrophils in blood and splenic cells. **(d)** Immune profiling of blood- and spleen- derived cells of the mice (first and second row respectively). For all cases, statistical significance was obtained by Kruskal-Wallis test followed by Dunn's uncorrected test. Immunophenotyping was performed in zombie-negative cells (viable cells). N = 5-7 mice per group. Data are represented as violin plots showing all data points. *P* values < 0.05 are indicated in the respective graph. MFI geometric mean fluorescent intensity

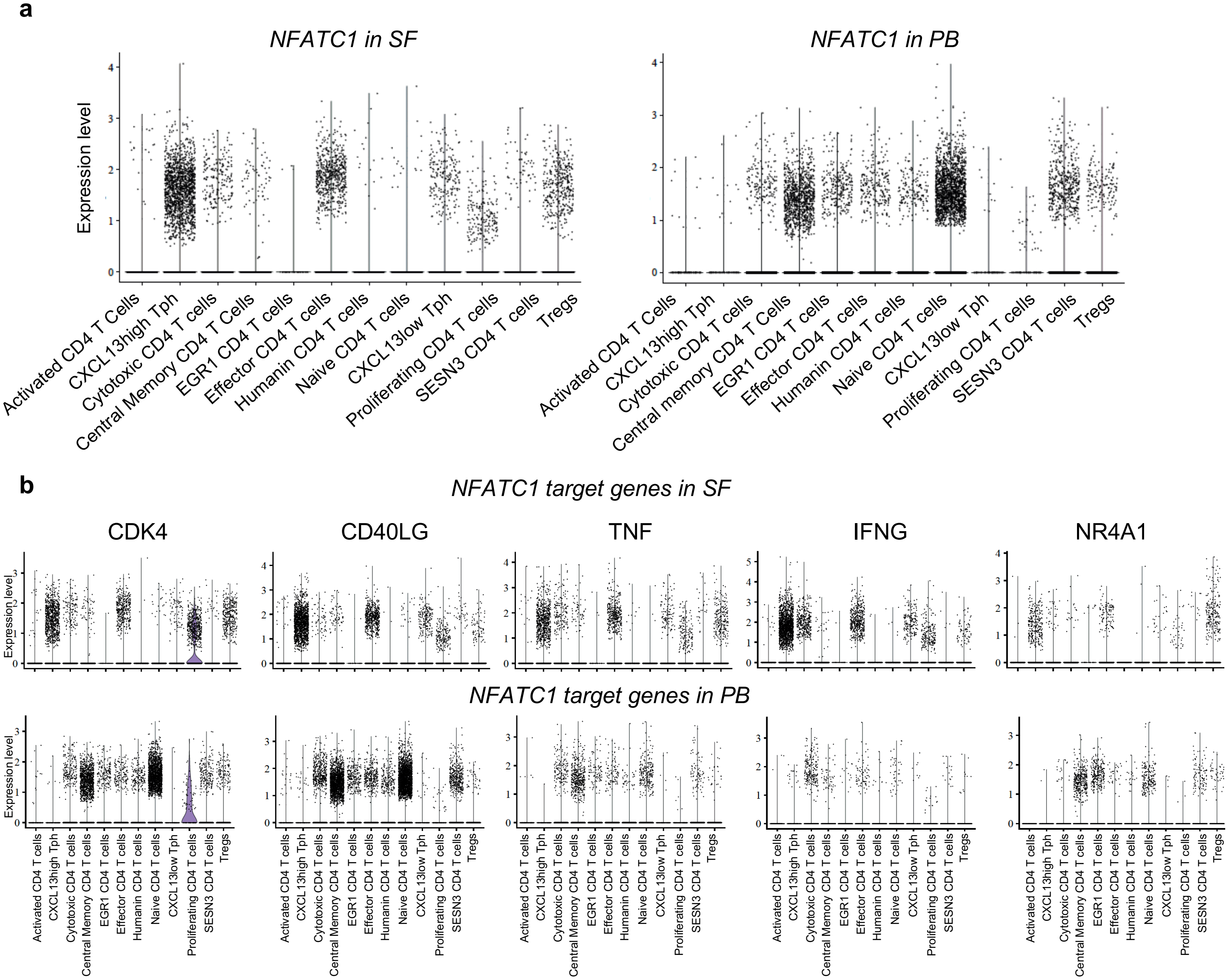


**Supplementary Figure 13.**

***NFATC1* and target genes expression across T-cell subsets in RA. (a)** Violin plots showing the expression of *NFATC1* in the different $CD4^+$ T-cell subsets in SF (left panel) and PB (right panel). **(b)** Violin plots showing the expression of *NFATC1* target genes in the different $CD4^+$ T-cell subsets in SF (upper panel) and PB (lower panel). RA rheumatoid arthritis, SF synovial fluid, PB peripheral blood

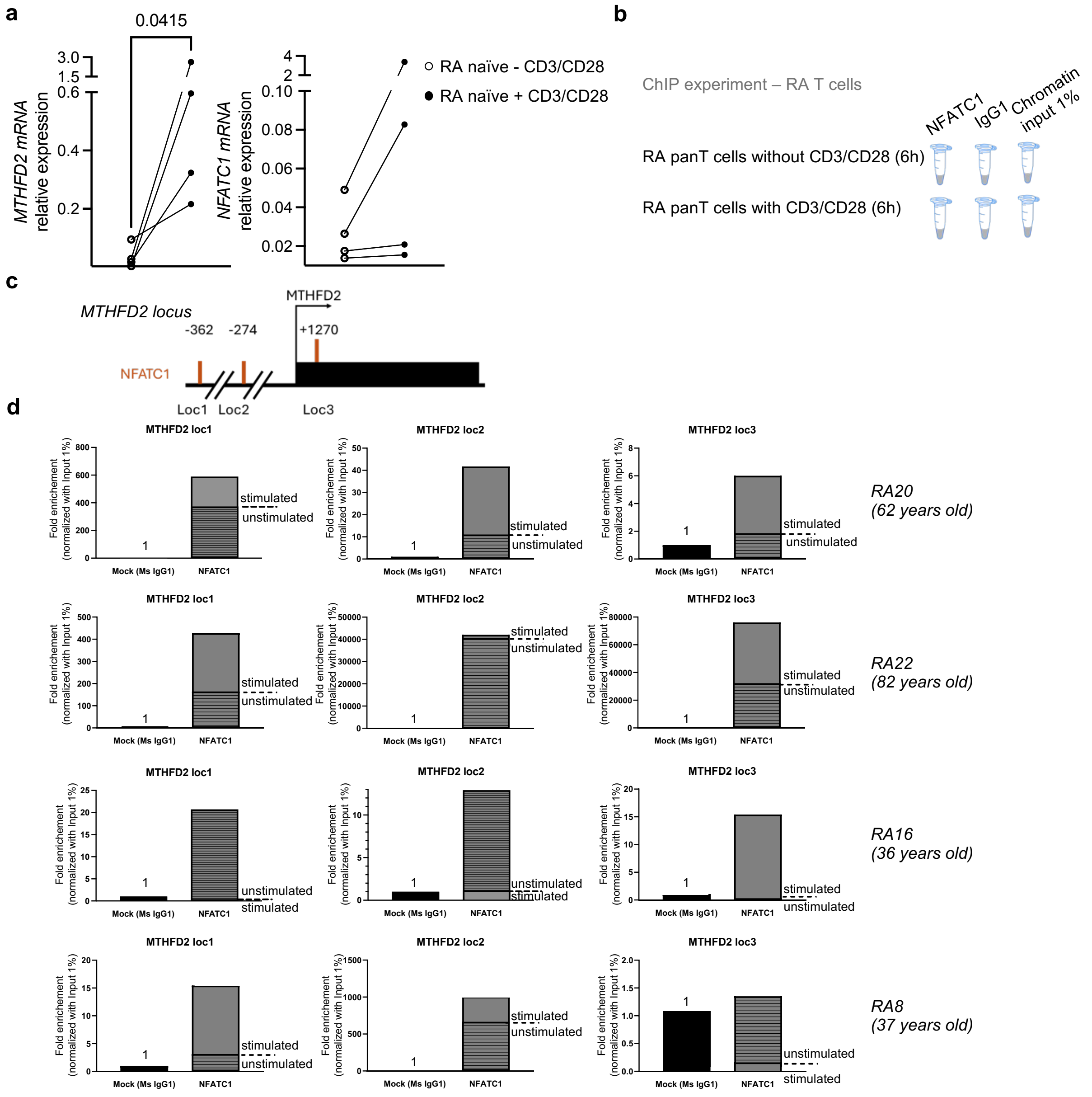


**Supplementary Figure 14.**

**ChIP analysis of RA T cells reveals strong binding of NFATc1 transcription factor on different genomic loci of *MTHFD2*.** **(a)** Pan T cells were isolated from the peripheral blood of treatment-naïve RA patients (n = 4) using a magnetic bead-based approach and cultured in the presence or absence of CD3/CD28 stimulatory beads for 6 hours. The relative expression levels of *MTHFD2* and *NFATC1* mRNA were then measured with real-time RT-qPCR (normalized to *ACTB*). Statistical significance was obtained by ratio paired *t* test. **(b)** Schematic representation of chromatin immunoprecipitation experiments (ChIP). ChIP experiments were performed using anti-NFATc1 antibody or a control antibody (IgG1) in chromatin isolated from pan T cells cultured for 6 hours in the presence or absence of CD3/CD28 beads and derived from treatment-naïve RA patients (n = 4). **(c)** Schematic representation of the *MTHFD2* gene locus around the transcription start site (TSS, arrow; coding region, black box). Numbers above the schematic drawing denote

the distance from the TSS and the predicted binding sites of NFATc1 (EPD database). **(d)** ChIP analysis of NFATc1 binding sites (loc1, loc2, loc3) at the *MTHFD2* gene locus. ChIP assays were performed using an anti-NFATc1 antibody or a control IgG1 antibody in chromatin isolated from unstimulated or CD3/CD28 *ex vivo* stimulated pan T cells derived from treatment-naïve RA patients (n = 4). Each graph depicts the fold enrichment of NFATc1 binding in unstimulated (striped bars) and stimulated (solid bars) cells. The numerical value on each bar represents the fold enrichment for stimulated cells. A binding site was considered valid only if the fold enrichment in stimulated cells exceeded that in unstimulated cells. RA rheumatoid arthritis, ChIP chromatin immunoprecipitation

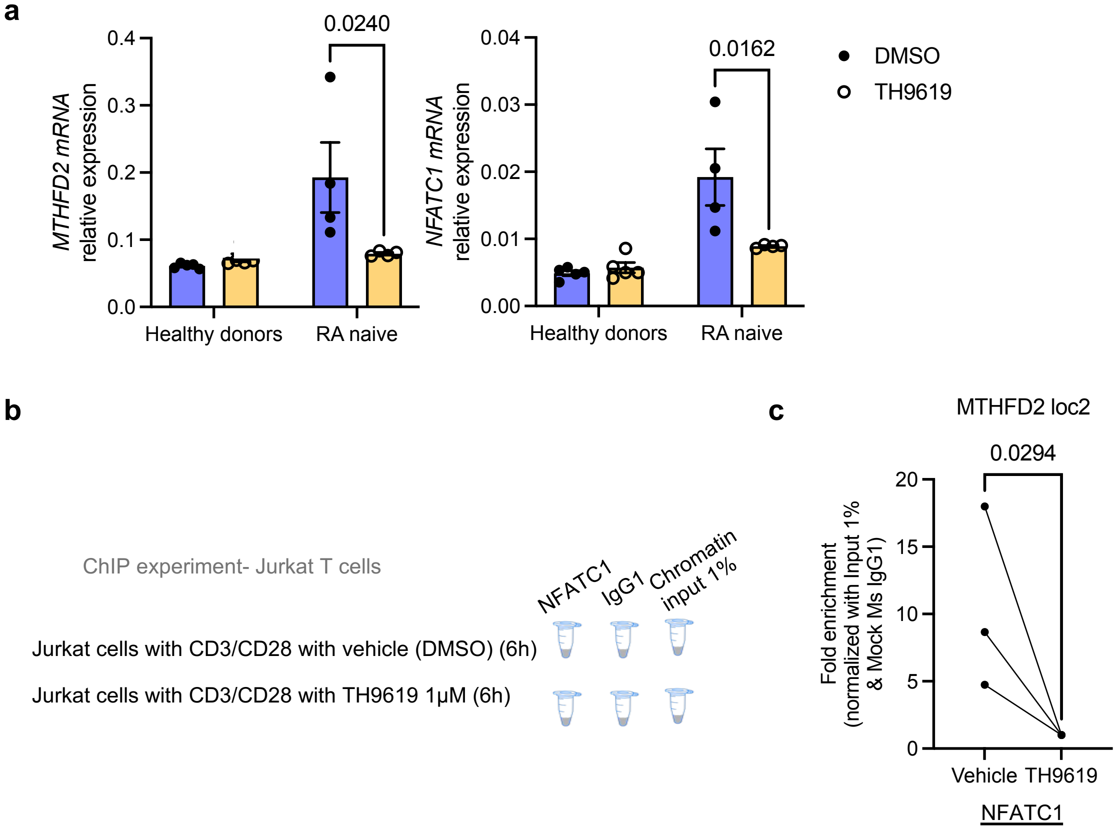


**Supplementary Figure 15.**

**TH9619 treatment inhibits NFATc1 binding to the *MTHFD2* genomic region. (a)** Pan T cells derived from the peripheral blood of healthy donors and treatment-naïve RA patients (n = 4 per group) were cultured in the presence of CD3/CD28 stimulatory beads and TH9619 (1 μM) or vehicle (DMSO) for 24 hours. The relative expression levels of *MTHFD2* and *NFATC1* mRNA were then measured with real-time RT-qPCR (normalized to *ACTB*). Statistical significance was obtained by two-way ANOVA. Data are represented as mean ± SEM. **(b)** Schematic representation of chromatin immunoprecipitation experiments (ChIP). ChIP experiment was performed using anti-NFATc1 antibody or a control antibody (IgG1) in chromatin isolated from Jurkat cells cultured for 6 hours with CD3/CD28 beads in the presence or absence of TH9619 (1 μM). **(c)** ChIP analysis of NFATc1 binding site (loc2) at the *MTHFD2* gene locus. ChIP assay was performed

using an anti-NFATc1 antibody or a control IgG1 antibody in chromatin isolated from CD3/CD28 stimulated Jurkat cells treated with TH9619 (1 µM) or vehicle (DMSO). N = 3 independent ChIP experiments are depicted. Statistical significance was obtained by ratio paired *t* test. *P* values < 0.05 are indicated in the respective graph. RA rheumatoid arthritis, ChIP chromatin immunoprecipitation

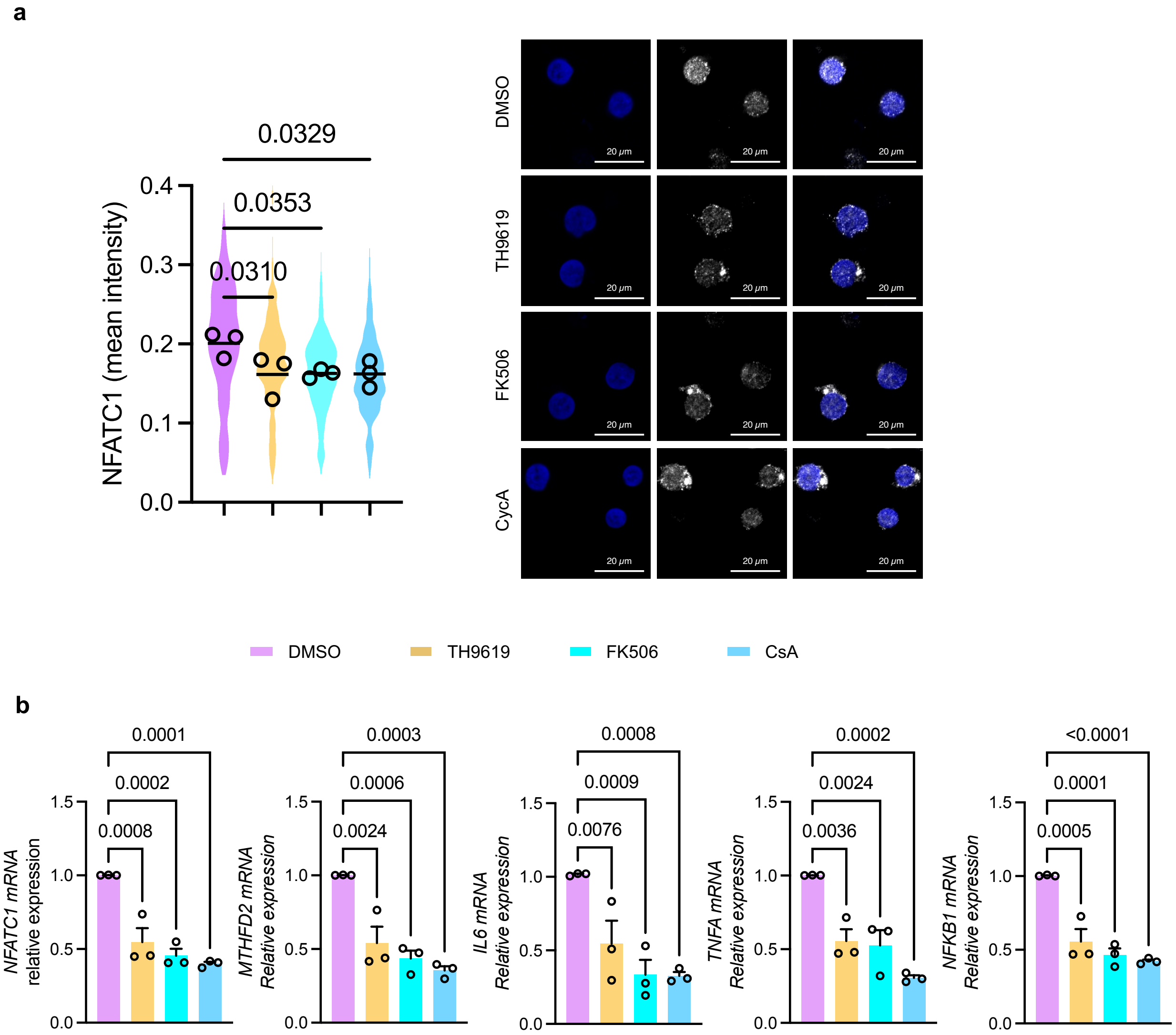


**Supplementary Figure 16.**
**Blocking NFATc1 nuclear translocation with calcineurin inhibitors recapitulates the effects of TH9619. (a)** Immunofluorescence microscopy analysis for fluorescence intensity of NFATc1 protein in Jurkat cells. Cells were cultured with CD3/CD28 stimulatory beads and the indicated compounds for 24 hours. Results are presented as SuperPlots where the circles represent the different individuals, and the violin plots represent the total of the mean fluorescence intensity (MFI) per cell. **(b)** Relative expression levels of *NFKB1* mRNA measured with real-time RT-qPCR (normalized to *ACTB* and DMSO group) following culture of Jurkat cells with CD3/CD28 stimulatory beads and the indicated compounds for 24 hours. Data are represented as mean ± SEM.

For the analyses, at least 200 cells per group (corresponding to a minimum of four different fields of each well) were observed for each marker under confocal microscopy. For all cases, statistical significance was obtained by one-way ANOVA. $P$ values < 0.05 are indicated in the respective graph. FK506 Tacrolimus, CsA Cyclosporin A

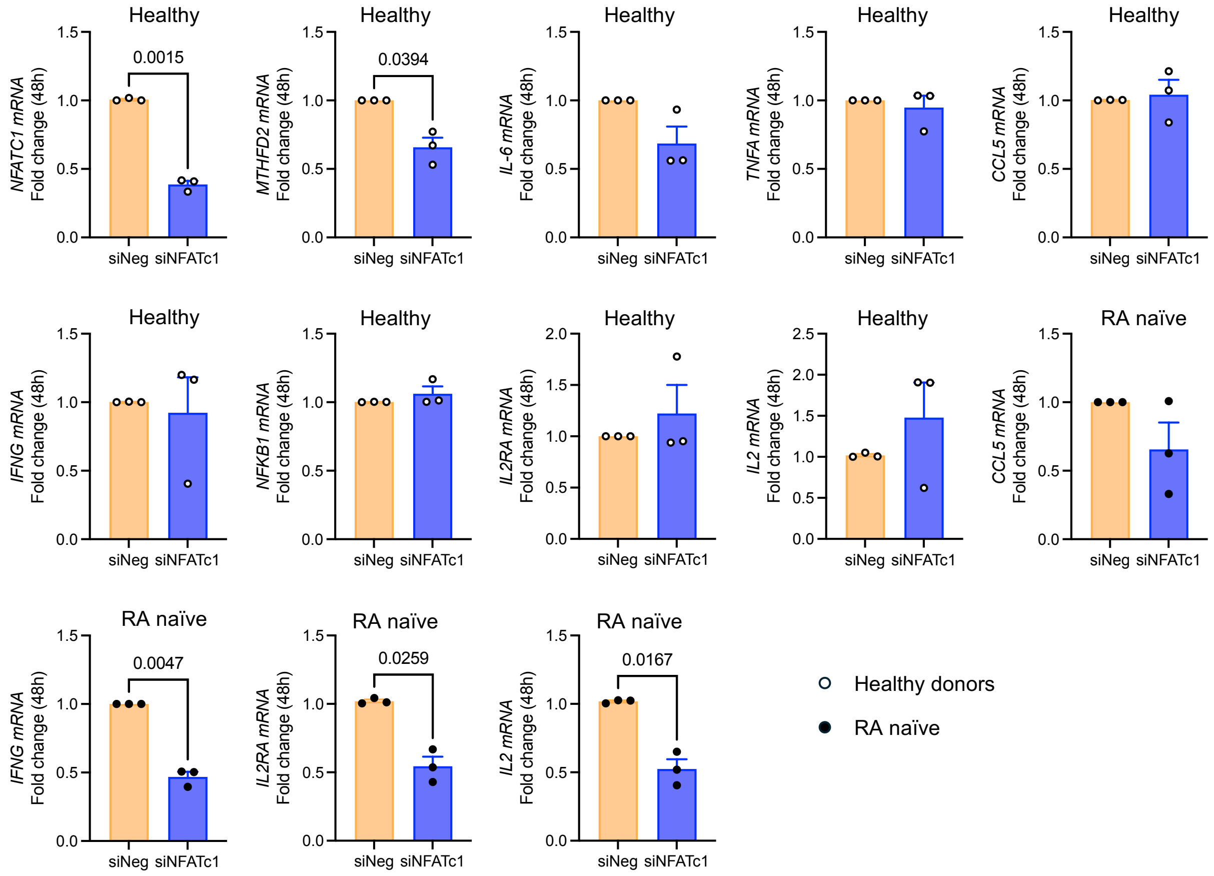


**Supplementary Figure 17.**
**NFATc1 targeting alters NFκB signaling in RA but not in healthy T cells.** Relative expression levels of *NFATc1, MTHFD2, IL6, TNF, CCL5, IFNG*, *NFKB1, IL2RA* and *IL2* mRNA of siNFATc1 and siNeg conditions in healthy or treatment-naïve RA pan T cells isolated from the peripheral blood (n = 3 individuals per group) measured with quantitative real-time RT-qPCR (normalized to *ACTB* and siNeg group). Data are represented as mean ± SEM. Statistical significance was obtained by paired $t$ test. $P$ values < 0.05 are indicated in the respective graph. RA rheumatoid arthritis

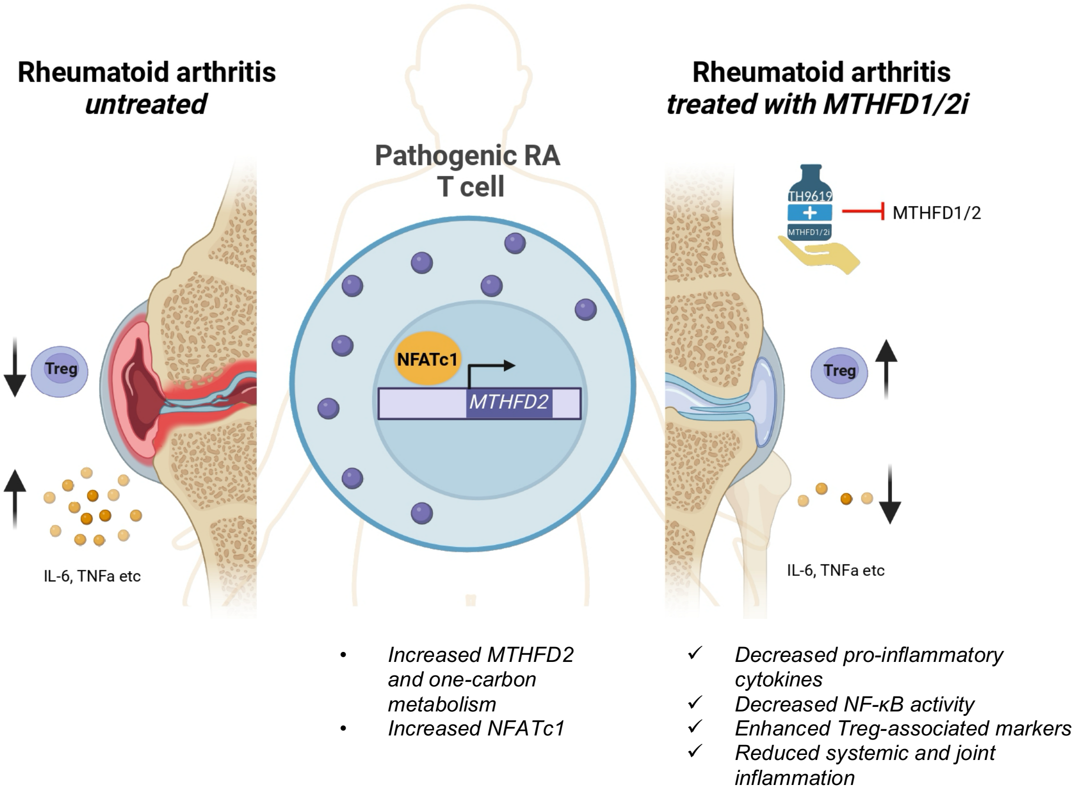


**Supplementary Figure 18.**

**Working Model.** In untreated rheumatoid arthritis, pathogenic T cells show elevated expression of one-carbon metabolism and MTHFD2 regulated by NFATc1, which correlates with increased inflammatory burden in affected tissues such as the synovium. This is accompanied by reduced regulatory T cell (Treg) function and elevated levels of pro-inflammatory cytokines, including IL-6 and TNFα. Treatment with the MTHFD1/2 inhibitor TH9619 disrupts the NFATc1-MTHFD2 interaction, leading to reduced inflammation by suppressing pro-inflammatory cytokine production, decreasing NF-κB activity and enhancing Treg-associated activity. Created with BioRender.com.

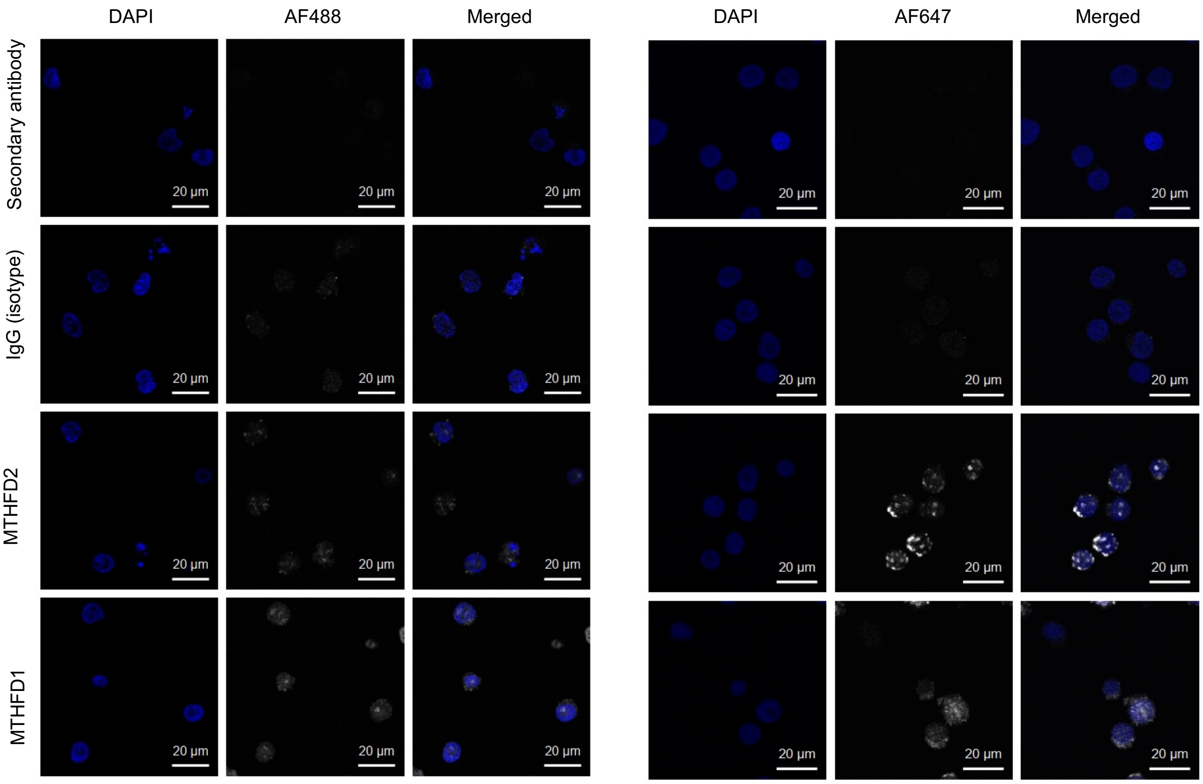


**Supplementary material.**

**Validation of MTHFD2 and MTHFD1 antibody specificity by isotype and secondary antibody control staining.** Fresh Jurkat cells were fixed with 4% paraformaldehyde for 15 minutes at room temperature and washed twice with DPBS. Cells were then blocked and permeabilized with 1% bovine serum albumin (BSA) in DPBS containing 0.1% Triton X-100 (blocking buffer) for 1 hour at room temperature. Cells were incubated with primary antibodies diluted in blocking buffer overnight at 4 °C, followed by secondary antibody incubation for 1 hour at room temperature in the dark. Finally, cells were resuspended in mounting medium (ibidi, Cat. 50001) and seeded in PhenoPlate™ 96-well plates (Revvity, Cat. 6055302). Imaging was performed using a Nikon AX R confocal microscope. Primary antibodies: MTHFD2 (Cell Signaling Technology, Cat. 41377S; 1:200), MTHFD1 (Atlas Antibodies, Cat. HPA000704; 1:230), Rabbit mAb IgG Isotype Control (Cell Signaling Technology, Cat. 3900S; 1:300). Secondary antibodies: Alexa Fluor 647 anti-rabbit (Invitrogen, Cat. A-31573; 1:300) and Alexa Fluor 488 anti-rabbit (Invitrogen, Cat. A-11008; 1:500).

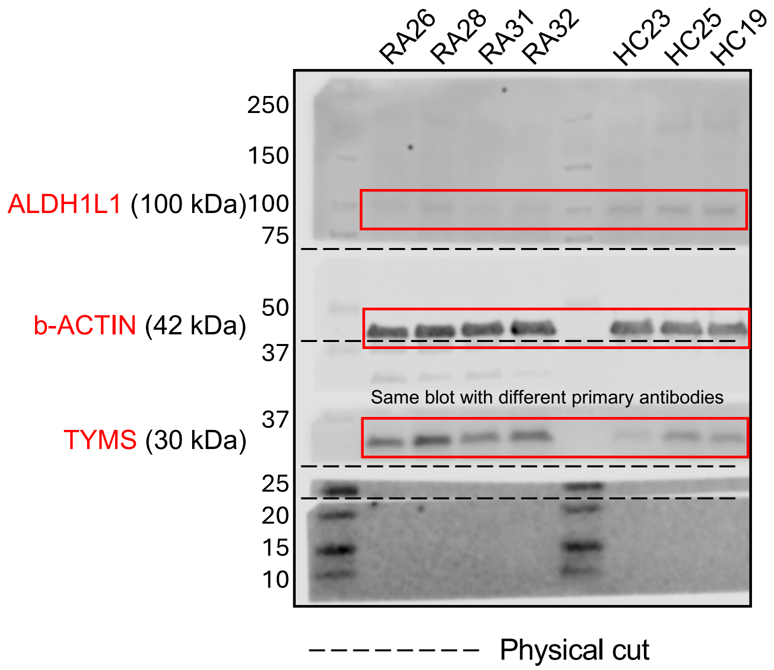


**Uncropped membranes for Supplementary Figure 2**

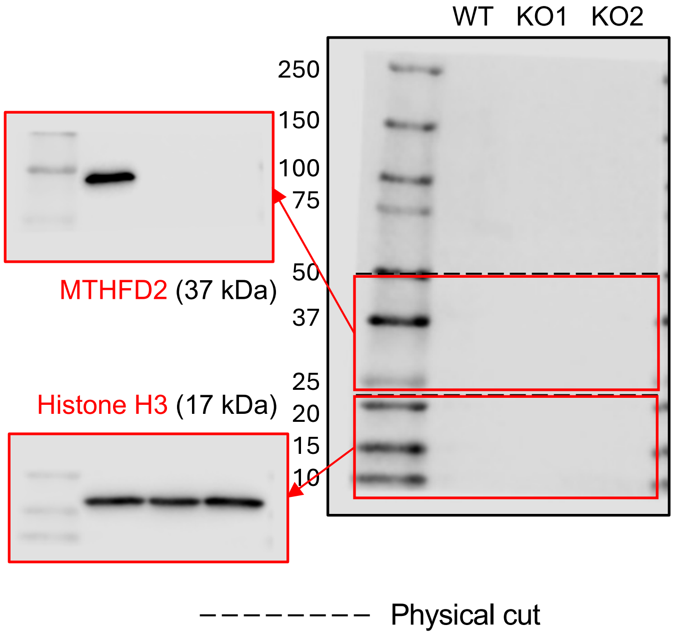


**Uncropped membranes for Supplementary Figure 8a**

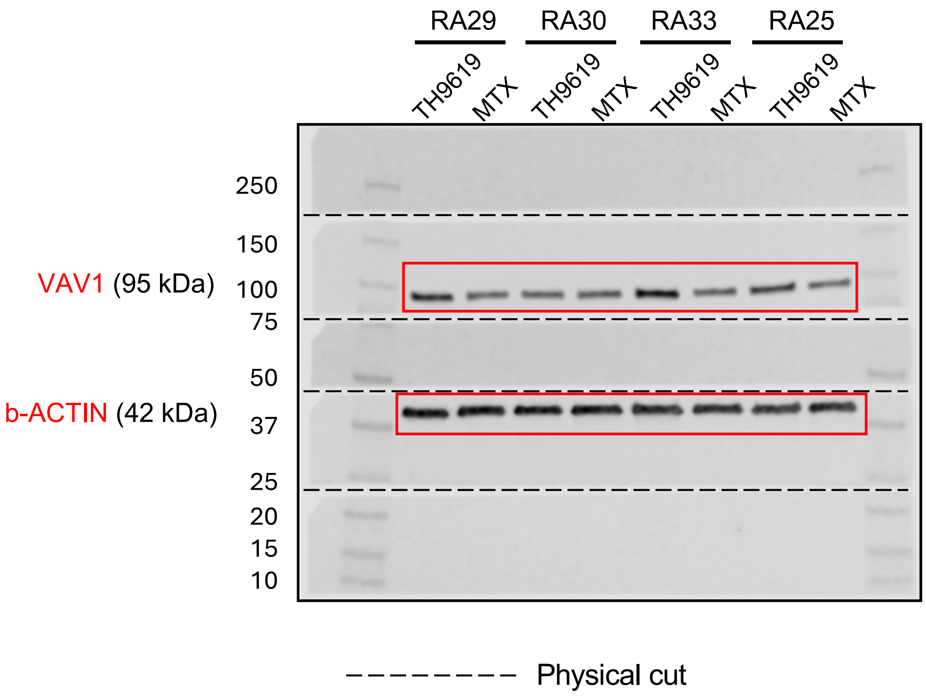


**Uncropped membranes for Supplementary Figure 9b**

**Supplementary material.**
**Uncropped Western blot images**
